# *Mafb* deficiency in myeloid cells increases susceptibility to *Mycobacterium tuberculosis* infection in mice

**DOI:** 10.3389/fimmu.2025.1660933

**Published:** 2026-01-23

**Authors:** Haruka Hikichi, Hajime Nakamura, Shiho Omori, Shintaro Seto, Minako Hijikata, Michito Hamada, Satoru Takahashi, Naoto Keicho

**Affiliations:** 1 Department of Pathophysiology and Host Defense, The Research Institute of Tuberculosis, Japan Anti-Tuberculosis Association, Tokyo, Japan; 2 Department of Basic Mycobacteriosis, Nagasaki University Graduate School of Biomedical Sciences, Nagasaki, Japan; 3 Laboratory Animal Resource Center in Transborder Medical Research Center, and Department of Anatomy and Embryology, Institute of Medicine, University of Tsukuba, Ibaraki, Japan; 4 Center for Medical Sciences, Ibaraki Prefectural University of Health Sciences, Ibaraki, Japan; 5 Vice Director, The Research Institute of Tuberculosis, Japan Anti-Tuberculosis Association, Tokyo, Japan

**Keywords:** conditional knockout mouse, host defense, MAFB, mRNAsequencing, *Mycobacterium tuberculosis*

## Abstract

v-Maf avian musculoaponeurotic fibrosarcoma oncogene homolog B (*MAFB*) is a candidate gene associated with early tuberculosis onset identified by a genome-wide association study. Here, we investigated the role of *Mafb* in susceptibility to *Mycobacterium tuberculosis* (*Mtb*) infection in myeloid-specific *Mafb*-knockout (*Mafb*-cKO) mice*. Mtb* infection was performed both *in vitro* using bone marrow-derived macrophages (BMMs) from *Mafb*-cKO mice and *in vivo* in *Mafb*-cKO mice. The absence of *Mafb* promoted *Mtb* proliferation in BMMs. RNA sequencing (RNA-seq) revealed activation of the metabolic process and impairment of the response to type I interferons (IFNs) in *Mtb*-infected BMMs from *Mafb*-cKO mice, which conforms to our previous findings in *Mtb*-infected human macrophages with *MAFB* knockdown. *Mafb* deficiency increased mortality and bacterial burden in the lungs and spleens during *Mtb* infection in mice. RNA-seq revealed weakened leukocyte or lymphocyte chemotaxis in *Mtb*-infected *Mafb*-cKO mouse lungs. Flow cytometry demonstrated an alteration in the proportion of immune cells in *Mtb*-infected mouse lungs due to *Mafb* deficiency. Together, *Mafb* in myeloid cells is involved not only in the functional antibacterial process of macrophages but also in immune cell recruitment in the lungs, thereby contributing to host defense against *Mtb* infection.

## Introduction

Tuberculosis (TB), caused by *Mycobacterium tuberculosis* (*Mtb*) infection, has resurged as the leading infectious disease, with 8.2 million newly diagnosed cases and 1.2 million deaths in 2023 alone (1). It is estimated that a quarter of the global population harbors a latent *Mtb* infection, characterized by the presence of the pathogen without symptoms. Individuals with latent infection have a lifetime risk of 5-10% of developing active TB, a risk that is substantially increased under conditions of immunosuppression, including HIV coinfection, malnutrition, or tobacco use. Therefore, management of latent *Mtb* infection, including early diagnosis, preventive therapy, or treatment, is critical to prevent further transmission and to ultimately achieve global TB elimination (2). Several studies have attempted to estimate the activation risk based on gene signatures or transcriptional biomarkers (3). Notably, identifying host factors that determine TB susceptibility is essential for understanding disease trajectory and accelerating drug and vaccine developments.

To date, numerous genome-wide association studies (GWASs) have been conducted to investigate the host genetic factors in TB susceptibility. However, only a few associations have proven reproducibility owing to the modest population sizes, variability in phenotyping across studies, population-specific effects, or complex population structures under certain high-burden settings (4). A meta-analysis combining two GWASs in Thai and Japanese populations did not replicate the association of 25 selected single-nucleotide polymorphisms (SNPs) (5). However, the age-stratified analysis from the same dataset revealed a significant locus on chromosome 20q12 linked to the younger onset group. This locus is located approximately 450-kb upstream of *v-maf avian musculoaponeurotic fibrosarcoma oncogene homolog B* (*MAFB*). Early-onset of TB implies the relatively sooner development after exposure to *Mtb*. The GWAS result suggests that *MAFB* plays a role in the host immunity toward controlling *Mtb* infection. With this background, we investigated the role of *MAFB* as a promising candidate gene involved in TB susceptibility.

**Figure 1:**
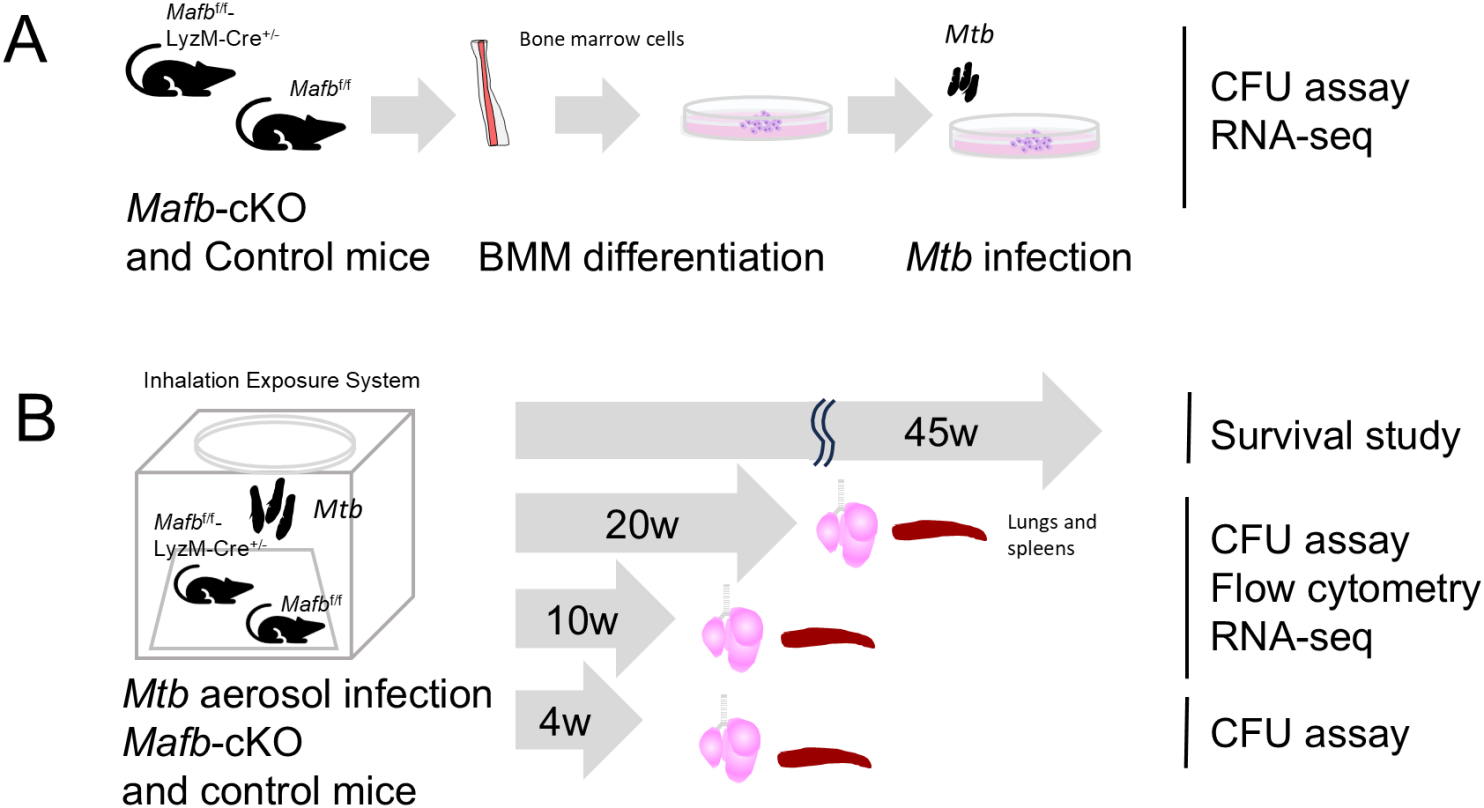
Schematic overview of the study design. The diagram illustrates the experimental timeline of bone marrow-derived macrophages (BMMs) from *Mafb*^f/f^::LysM-Cre^+/-^ (*Mafb*-cKO) and *Mafb*^f/f^ (control) mice with *Mycobacterium tuberculosis* (*Mtb*) infection **(A)** and *Mtb* aerosol infection of *Mafb*-cKO and control mice **(B)**. Subsequent analyses included such as CFU assay, flow cytometry, and mRNA sequencing (RNA-seq).

MAFB belongs to the large Maf family of transcription factors characterized by a conserved basic leucine zipper (bZip) enabling specific DNA binding to Maf-recognition elements (MAREs) (6). *Mafb* plays a crucial role in the organogenesis of various organs and in maintaining macrophage homeostasis (7). In the context of immune regulation and infectious disease, *MAFB* has been reported to control antiviral response and macrophage polarization (8, 9). Previously, we investigated the function of *MAFB* in *Mtb*-infected human macrophages to explore the biological mechanism underlying *MAFB* in macrophages (10). Our gene knockdown (KD) experiments revealed that MAFB regulates the gene expression related to interferon (IFN) responses in *Mtb*-infected macrophages. In the present study, we investigated the role of *MAFB*, particularly in disease outcomes and dynamic immune cell interactions in organisms by using myeloid-specific *Mafb*-knockout (*Mafb*-cKO) mice (11) (**Figure 1**). We monitored the survival and bacterial burden in the murine organs and found that *Mafb*-cKO mice had higher mortality and bacterial burden during the *Mtb* infection. RNA sequencing (RNA-seq) of *Mtb*-infected *Mafb*-cKO mouse lungs revealed a disrupted chemotaxis. These results were consistent with altered immune cell populations in the lungs of *Mtb*-infected *Mafb*-cKO mice. Taken together, this study highlights *MAFB* as an important gene in macrophages that contributes to protective immunity against *Mtb* infection.

## Materials and methods

### Ethics statement

Animal experiments in this study were approved by the Animal Care and Use Committee of the Research Institute of Tuberculosis (RIT) (permit number: No. 2021-04). Animals were treated in accordance with the ethical guidelines of RIT. The endpoints were set to determine whether the mice were imminently dying of *Mtb* infection and/or required compassionate euthanasia: bodyweight loss >20% of the initial bodyweight at the time of infection.

### Mice

Macrophage-specific *Mafb* conditional-knockout (*Mafb*^f/f^::LysM-Cre^+/+^ or *Mafb*^f/f^::LysM-Cre^+/-^, *Mafb*-cKO) and *Mafb*^f/f^ control mice were used (11). *Mafb*-cKO and control mice were maintained under pathogen-free conditions in a laminar-flow facility. Wild-type (WT) C57BL/6J mice were obtained from The Jackson Laboratory Japan, Inc. Specific pathogen-free status was verified by testing sentinel mice housed within the colony.

### *Mtb* culture

The *Mtb* strain Erdman was used and stored as previously described (12–14). For determining the bacterial burden in macrophages, the infected cells were lysed with PBS containing 0.1% SDS. Infected lungs or spleens were homogenized using a ShakeMaster Neo (Bio Medical Science). The resulting cell lysates or homogenates were serially diluted and plated in duplicate on 7H10 or 7H11 agar plates supplemented with 10% Middlebrook OADC (BD Bioscience) and 0.5% glycerol. *Mtb* colony-forming units (CFUs) were determined by calculating the mean CFU count from the two plates at each dilution.

### Bone marrow-derived macrophage isolation

BMMs were differentiated as previously described (15), with some modifications. Briefly, bone marrow was isolated from the hind legs of *Mafb*-cKO and control mice (6 weeks), washed, and suspended into a single cell by passing through a cell strainer. The bone marrow cells were then incubated at a concentration of 2 × 10^6^ cells/mL in DMEM (Sigma-Aldrich) supplemented with 10% inactivated-fetal bovine serum (FBS, JRH Biosciences Inc.) and 10% of L929-conditioned medium in a 12-well plate for 7 days. Differentiated macrophages in DMEM containing 10% FBS were infected with *Mtb* at a multiplicity of infection (MOI) of one. At one day postinfection (p.i.), BMMs were collected for mRNA sequencing (mRNA-seq). At 1, 3, and 7 days p.i., the number of the intracellular bacteria within BMMs was determined by CFU.

### Cytotoxicity test

Cytotoxicity was evaluated colorimetrically by measuring lactate dehydrogenase (LDH) released into the culture supernatant using a Cytotoxicity LDH Assay Kit (Dojindo). Briefly, BMMs from control or *Mafb*-cKO mice were infected with *Mtb* at an MOI of 1, and the LDH assay was performed at 1, 3, and 7 days p.i. The optical density at 490 nm (OD) was measured using a Varioskan LUX multimode microplate reader (Thermo Scientific). For each condition, the mean OD of four replicate wells was calculated, the background (medium) value was subtracted, and cytotoxicity was expressed as a percentage of the maximal reaction obtained by complete cell lysis. Cytotoxicity (%) = (sample OD − medium OD)/(maximal reaction OD − medium OD) × 100.

### *Mtb* infection in mice

The experimental mice (age: 6–10 weeks) were transferred to a biosafety level 3 animal facility at RIT. The mice were infected with *Mtb* via the aerosol route using an inhalation exposure system (Glas-Col). This method routinely gave *Mtb* infection at 100–200 CFU per lung one day p.i.

### Survival study

WT and *Mafb*-cKO mice infected with *Mtb* were monitored for 315 days. The mice that survived throughout the experiments or met the endpoint were euthanized by exsanguination under anesthesia with 0.75 mg/kg of medetomidine, 4.0 mg/kg of midazolam, and 5.0 mg/kg of butorphanol *via* the intraperitoneal route (16). Survival probabilities between the two groups were analyzed using Kaplan–Meier analysis and the log-rank test. The body weight of infected mice was monitored during the infection.

### mRNA-seq

mRNA-seq of *Mtb*-infected BMMs or whole lung lobes of *Mtb*-infected mice was performed as previously described (14). Briefly, infected BMMs or whole lung lobes were homogenized with TRIzol Reagent (Invitrogen), followed by RNA purification using an RNeasy Mini kit (Qiagen). Total RNA qualified and quantified by a 4150 TapeStation system (Agilent) and a Qubit 4 Fluorometer (Invitrogen), respectively, was subjected to construct cDNA libraries using NEBNext^®^ Poly(A) mRNA Magnetic Isolation Module (New England Biolabs) and NEB Next Ultra™ II DNA Library Prep Kit for Illumina (New England Biolabs). All the cDNA libraries were examined for quality using a 4150 TapeStation system and quantified with a Qubit 4 Fluorometer. The libraries were sequenced with a NextSeq1000 system (Illumina).

### Data processing

For read-quality trimming, raw reads were processed with Trim Galore v0.6.7 (https://github.com/FelixKrueger/TrimGalore). The expressions of transcripts were estimated by Salmon v0.12.0 directly from the processed reads (17). Transcript-level expression data was then summarized into gene-level data by the R package tximport v1.30.0 (https://github.com/thelovelab/tximport) in R v4.3.3 (18). The analysis for differentially expressed genes (DEGs) was performed by edgeR v4.0.16 (19) using generalized linear models and quasi-likelihood tests (20). The DEGs were further utilized to perform Gene Ontology (GO) enrichment analysis to identify enriched BPs using the R package clusterProfiler v4.10 (21). To reduce redundancy among the identified GOBP categories, a simplification method in clusterProfiler was used. The gene concept network of the top 3 upregulated and downregulated GOBP categories was visualized by cnetplot in clusterProfiler. KEGG pathway enrichment analysis was conducted using ShinyGO v0.82 (22), an online gene set enrichment tool (http://bioinformatics.sdstate.edu/go/). Adapted KEGG pathway diagrams were visualized using Pathview v1.42.0 in R software (23). Pathway source: KEGG (https://www.kegg.jp). Gene set enrichment analysis (GSEA) was performed locally using the GSEA desktop application (Broad Institute, v4.2.3) with the WikiPathway gene sets (c2.cp.wikipathways.v2024.1.Hs.symbols.gmt) obtained from the Molecular Signature Database (MSigDB). MafB ChIP-seq peaks (GEO GSM1964739/SRA SRX1465586) (24) were downloaded via ChIP-Atlas (accessed 27 June 2025) (25) and compared with the DEGs identified in the present study. The ChIP-seq Atlas is accessible at https://chip-atlas.org/.

### Data availability

The sequencing data generated in this study were deposited in the DNA Data Bank of Japan under the BioProject accession number PRJDB20606.

### Quantitative reverse transcription PCR

Quantitative reverse transcription PCR (qRT-PCR) was performed as previously described (10) with minor modifications. Briefly, total RNA from BMMs or mouse lungs was reverse-transcribed into cDNA and subjected to qRT-PCR using a KAPA SYBR Fast qPCR kit (Roche) on a QuantStudio Pro 7 system (Invitrogen). The primers used in this study are listed in **Supplementary Table 1**. The threshold cycle (Ct) values of target genes were normalized to that of *Rplp1* and compared with the control group.

### Flow cytometry

Infected lung cells were obtained using the Lung Dissociation Kit (Miltenyi Biotec) according to the manufacturer’s instructions. Briefly, cell suspensions from the lungs were incubated with ACK buffer to lyse red blood cells. The cells were washed and diluted in MACS buffer (PBS supplemented with 2mM EDTA and 2% FBS) to achieve 1–3 × 10^6^ cells/mL. The cells were incubated with TruStain FcX™ PLUS (anti-mouse CD16/32) (Biolegend), followed by staining with antibodies against CD4, CD8, CD45R, CD3, SiglecF, CD64, CD11b, CD45, or Ly6G (BioLegend). The stained cells were then fixed with the fixation buffer (BioLegend) to inactivate infected *Mtb* for 24 h at 4°C. The cells were analyzed on a BD FACSLyric™ using analysis software BD FACSuite™ Application V1.4.0.7047 and FlowJo™ Software v10.10 (BD Biosciences).

### Histological analysis

Whole lung lobes from infected mice were fixed with 10% formalin in PBS for over 24 h at room temperature. Tissue sections were stained with hematoxylin and eosin (H&E). Immunohistochemistry (IHC) analysis was performed as previously described (26–28). Tissue sections were stained with anti-S100a9 (1:200, R&D Systems) and digitized using a NanoZoomer S60 slide scanner (Hamamatsu Photonics). The resulting IHC images were analyzed with QuPath (29) to perform cell detection followed by object-based classification within each annotated granuloma region to quantify S100a9^+^ cells.

### Fluorescence imaging of intracellular *Mtb*

BMMs from control and *Mafb*-cKO mice were grown on coverslips in 12-well plates and infected with DsRed-expressing *Mtb*. At 1, 3, and 7 days p.i., cells were fixed with 3% paraformaldehyde in PBS at 4°C for 24 h, washed three times with PBS, and mounted on microscope slides using Vectashield Antifade Mounting Medium with DAPI (Vector Laboratories). Fluorescence microscopy was performed using an Olympus IX81 microscope equipped with a DP74 camera (Olympus). DAPI and DsRed fluorescence images were merged, and the number of intracellular *Mtb* bacilli was quantified in ImageJ (version 1.54g) (30).

### ELISA

The concentrations of secreted MCP-1 and IP-10 from control and *Mafb*-cKO BMMs infected with *Mtb* were measured using the Mouse CCL2/JE/MCP-1 Quantikine SixPak 2nd Gen ELISA and the Mouse CXCL10/IP-10/CRG-2 DuoSet ELISA (R&D Systems), respectively. Culture supernatants were collected from infected macrophages at 1, 3, and 7 days p.i. and filtered through a 0.45-µm pore-size filter (Toyo Roshi Kaisha).

## Results

### *Mafb* deficiency on mycobacterial killing in macrophages

**Figure 2:**
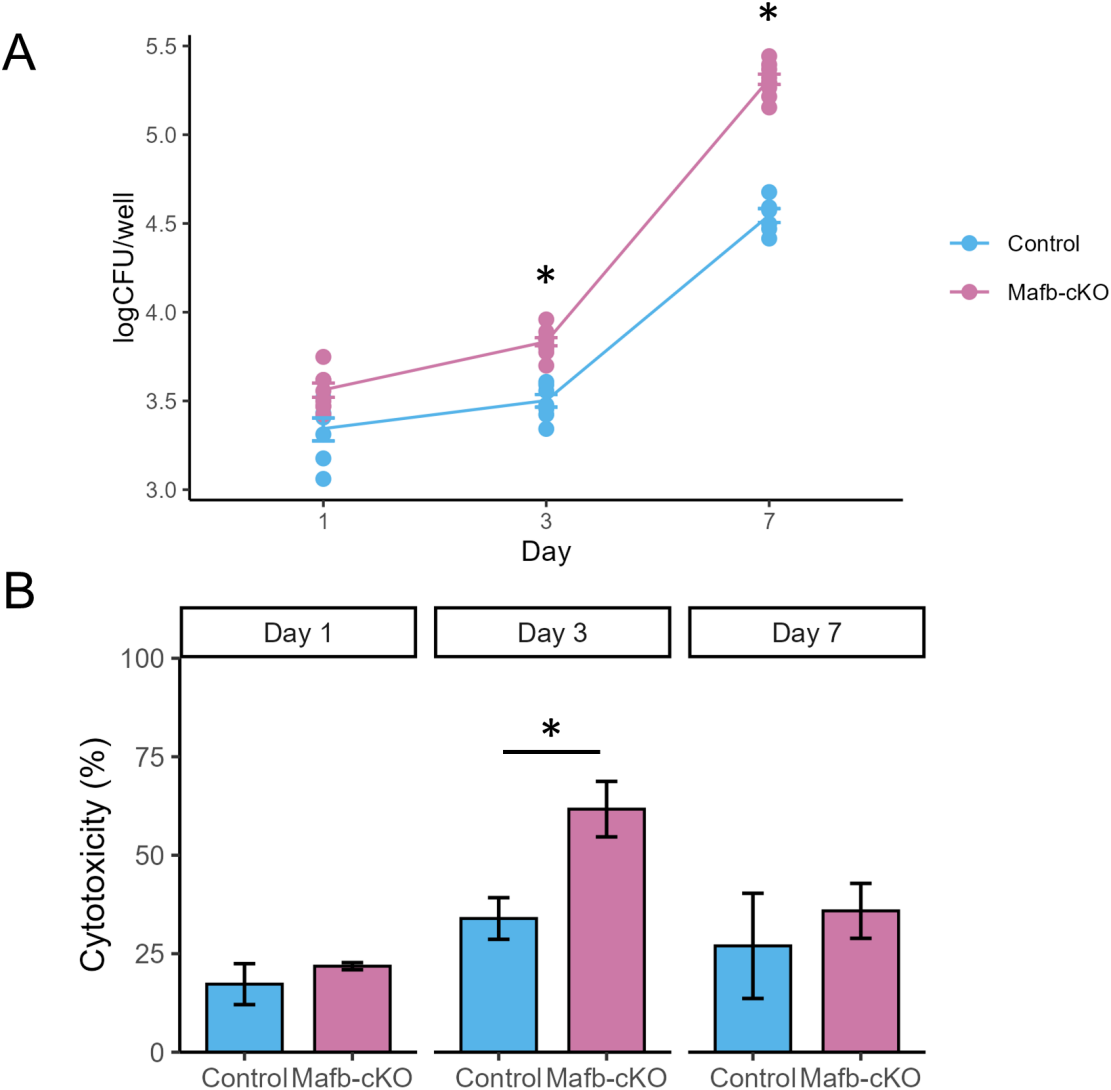
Mycobacterial proliferation on *Mafb*-deficient macrophages. **(A)** CFU assay. BMMs from *Mafb*-cKO and control mice were infected with *Mtb*. At 1, 3, and 7 days postinfection (p.i.), the numbers of the intracellular bacteria were determined by CFU assay (n = 6–10 wells per group at each time point). *P < 0.01 using Welch’s t-test, with Holm–Bonferroni correction applied for multiple comparisons. **(B)***Mtb*-induced cytotoxicity in BMMs. BMMS from *Mafb*-cKO or control mice were infected with *Mtb*. Lactate dehydrogenase (LDH) assay was performed at 1, 3, 7 days p.i. (n = 4 wells per group at each time point). *P < using Welch’s t-test.

In our previous study, we demonstrated impaired inflammatory responses in PMA-stimulated *MAFB*-knockdown THP-1 cells (*MAFB*-KD macrophages). However, no significant difference in bacterial burden was observed between *MAFB*-KD macrophages and control macrophages at 24 h or 48 h p.i., suggesting that the knockdown effect and/or the duration of infection was insufficient to detect intracellular bacterial proliferation (10). In this study, we investigated the effect of *Mafb* deficiency on mycobacterial proliferation in BMMs (**Figures 1A**, **2**). Using BMMs derived from *Mafb*-cKO or control mice, we compared *Mtb* proliferation within BMMs (**Figure 2A**) and *Mtb* infection-induced cytotoxicity (**Figure 2B**). We infected BMMs with *Mtb* at an MOI of one and monitored CFU and cytotoxicity at 1, 3, and 7 days p.i. We confirmed the depletion of *Mafb* expression in BMMs from *Mafb*-cKO mice by mRNA-seq (**Supplementary Figure 1**). At 3 and 7 days p.i., intracellular *Mtb* proliferation was significantly higher in *Mafb*-cKO BMMs, suggesting that the absence of *Mafb* transforms macrophages into a more permissive environment for *Mtb* proliferation. For *Mtb*-induced cytotoxicity, BMMs from *Mafb*-cKO also showed greater susceptibility at day 3 p.i., which aligns with the higher bacterial burden in *Mafb*-cKO BMMs. These results support the concept that host cell death accelerates intracellular *Mtb* growth (31).

### *Mafb*-cKO BMMs demonstrated functional changes in metabolic process and immune response during *Mtb* infection

**Figure 3:**
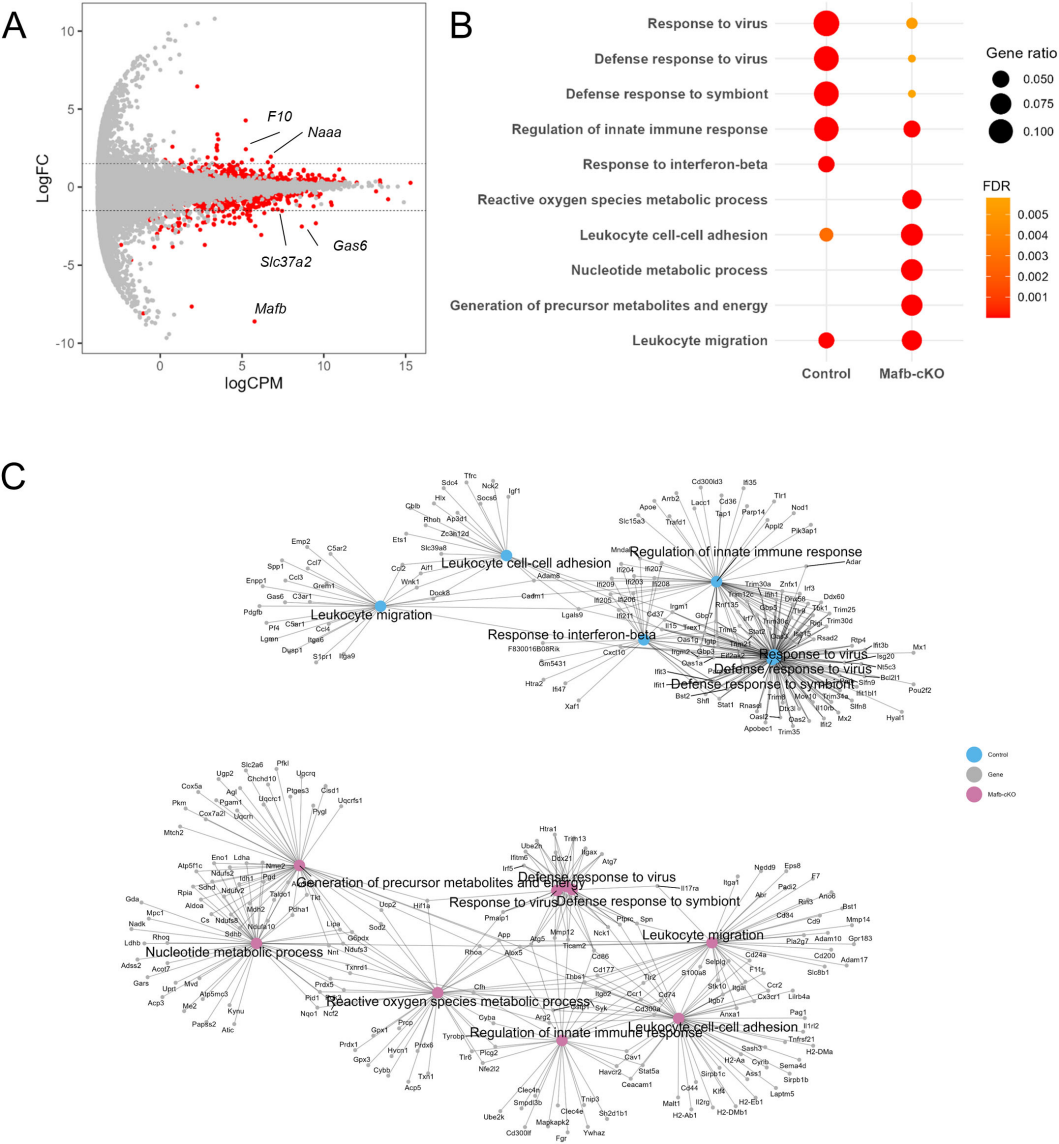
Transcriptomics of *Mtb*-infected *Mafb*-cKO BMMs. **(A)** mRNA sequencing (mRNA-seq) of *Mtb*-infected *Mafb*-cKO BMMs was performed (n = 4 per group). MA plot showing 1223 differentially expressed genes (DEGs) in *Mtb*-infected *Mafb*-cKO BMMs compared to those in *Mtb*-infected *Mafb*^f/f^ control BMMs, marked in red (*P* values adjusted using the false discovery rate (FDR) < 0.01). Each dot represents expressed genes in the sample. Log FC, log fold change. LogCPM, log count per million. **(B)** Gene Ontology (GO) analysis for upregulated or downregulated DEGs. Enriched GO biological process (BP) categories in *Mtb*-infected *Mafb*-cKO BMMs can be seen. The color of each dot represents FDR, and the size represents gene ratio. **(C)** Gene concept networks of the top 3 upregulated (Up) and downregulated (Down) GOBP categories in *Mtb*-infected *Mafb*-cKO BMMs. Upregulated GOBP categories are leukocyte cell–cell adhesion, reactive oxygen species metabolic process, and nucleotide metabolic process, colored in salmon pink. Downregulated GOBP categories are response to virus, defense response to symbiont, and regulation of innate immune response, as shown in blue.

When macrophages are exposed to *Mtb*, they internalize the bacteria, and *Mtb* begins adapting to the intracellular environment by 24 h p.i. During this period, macrophages undergo robust transcriptional changes, indicating active host–pathogen interactions (32). To investigate the transcriptional function of *Mafb* in *Mtb*-infected macrophages, we infected BMMs from *Mafb*-cKO and control with *Mtb* and conducted mRNA-seq at 24 h p.i. mRNA-seq comparing between *Mtb*-infected BMMs from *Mafb*-cKO and control mice identified 1223 DEGs (**Figure 3A**, **Supplementary Table 2**). GO analysis for BP (GOBP) identified 974 significantly enriched GOBP terms in 614 upregulated DEGs in *Mtb*-infected *Mafb*-cKO BMMs, including leukocyte cell–cell adhesion, reactive oxygen species (ROS) metabolic process, or nucleotide metabolic process (**Figure 3B**, **Supplementary Table 3**). In 609 downregulated DEGs, 493 significantly enriched GOBP terms were identified, including response to virus, defense response to symbiont, or regulation of innate immune response (**Figure 3B**). Some GOBP terms, such as leukocyte migration and response to virus, were shared between upregulated and downregulated DEGs.

**Figure 4:**
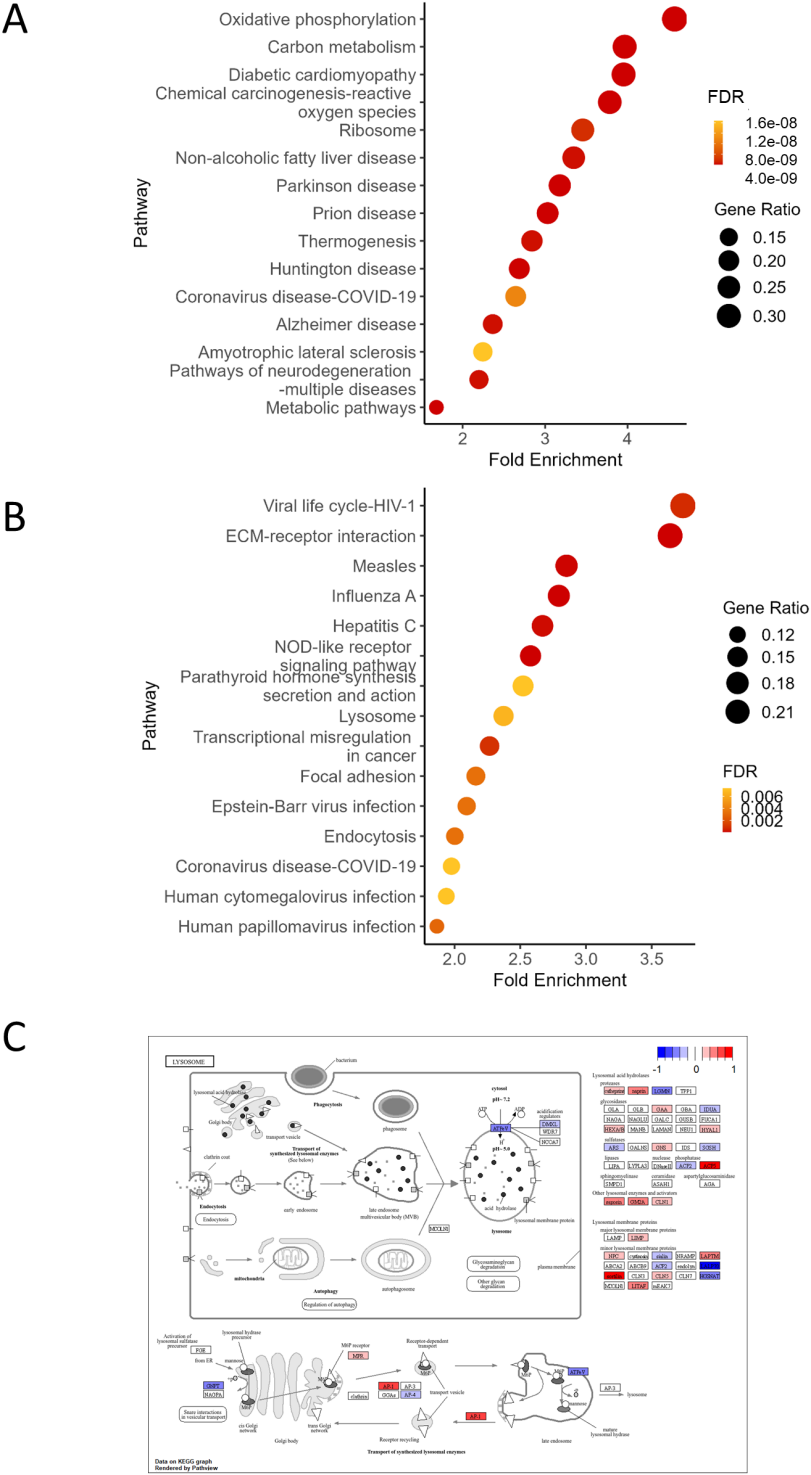
KEGG pathway enrichment analysis of DEGs in *Mtb*-infected *Mafb*-cKO BMMs. **(A, B)** KEGG pathway enrichment analysis was performed on upregulated DEGs **(A)** or downregulated DEGs **(B)** of *Mtb*-infected *Mafb*-cKO BMMs. **(A)** Among the upregulated DEGs, oxidative phosphorylation and chemical carcinogenesis-reactive oxygen species are enriched. **(B)** Among the downregulated DEGs, ECM-receptor interaction, NOD-like receptor-signaling pathway, and lysosome were enriched. The color of each dot represents FDR, and the size represents gene ratio. **(C)** The KEGG pathway diagram of the lysosome (mmu04142) is shown. Genes in the pathway are color-coded based on logFC in *Mtb*-infected *Mafb*-cKO BMMs (red) compared to those in *Mtb*-infected control BMMs (blue). Pathway map adapted from KEGG: https://www.kegg.jp/pathway/mmu04142.

**Figure 5:**
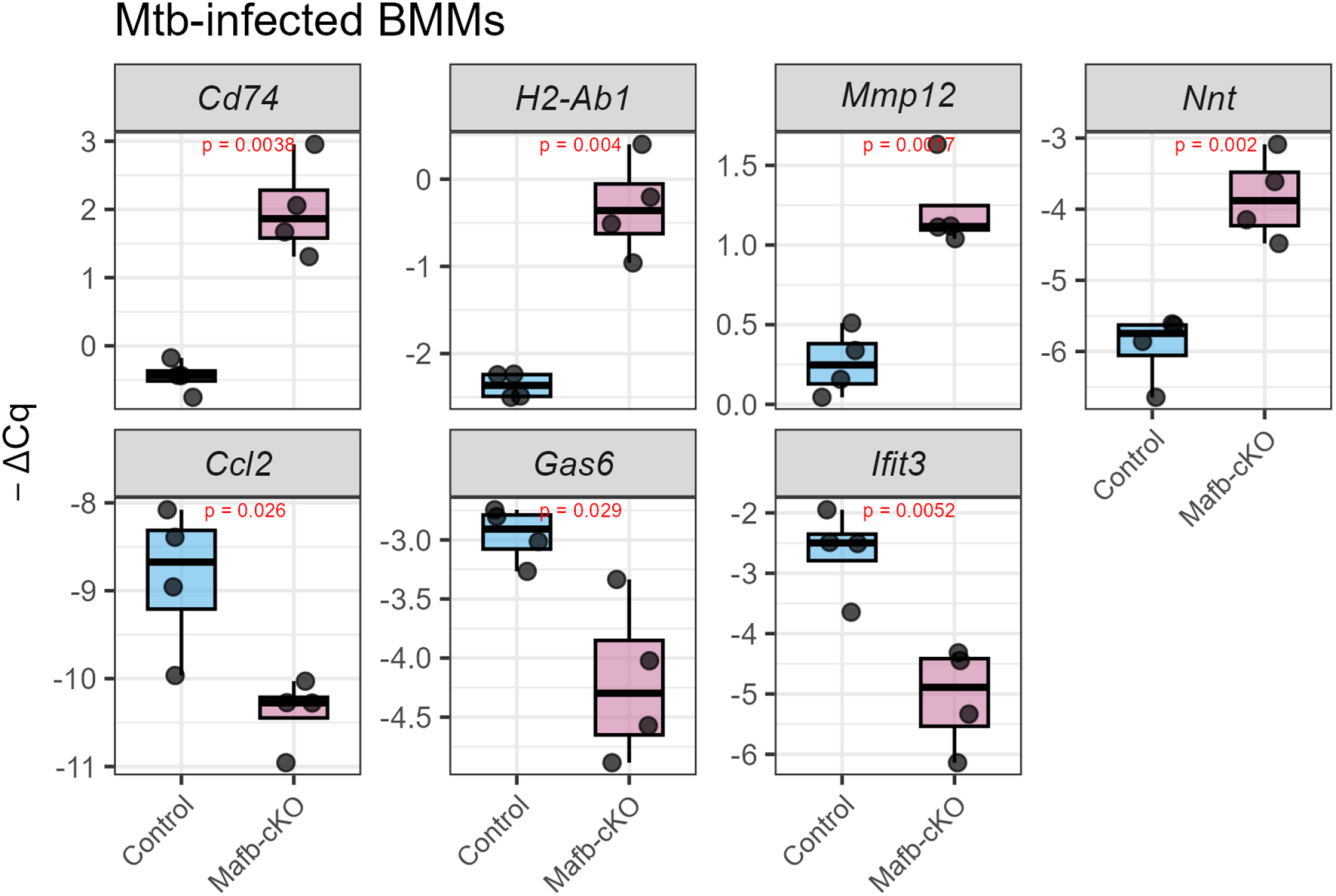
Validation of DEGs in *Mtb*-infected BMMs by quantitative reverse transcription PCR. Seven representative DEGs were selected for validation by quantitative reverse transcription PCR (qRT-PCR) (n= 4 per group). Upregulation of *Cd74*, *H2-Ab1*, *Mmp12*, and *Nnt*, and downregulation of *Ccl2*, *Gas6*, and *Ifit3* in *Mtb*-infected *Mafb*-cKO BMMs were confirmed.

To visualize the interactions of the genes annotated to each GO term, we constructed gene networks (**Figure 3C**). Upregulated genes annotated to leukocyte cell–cell adhesion are associated with adhesion molecules or integrins (e.g., *Itgb2*, *Itgb7*, *Itgal*), leukocyte surface receptors (e.g., *Ccr2*, *Cx3cr1*, *Cd74*), MHC molecules (e.g., *H2-Ab1*, *H2-Aa*), and immune modulation (e.g. *Sirpb1b*, *Arg2*, *Thbs1*), suggesting that the macrophages are in the state where they are actively participating in immune surveillance, cellular communication, and antigen presentation. Upregulated genes were also annotated to ROS metabolic process including ROS generation (e.g. *Cybb*, *Cyba*), ROS detoxification and antioxidant defense (e.g., *Prdx1*, *Nnt*), and oxidative stress modulation (e.g., *Thbs1*, *Rhoa*). The downregulated genes annotated to top significantly enriched GOBP terms were highly overlapped: RNA editing and modification (e.g., *Apobec1*, *Adar*, *Ifi204*), innate immune sensors and IFN-stimulated genes (ISGs) (e.g., *Ifit1*, *Ifit2*, *Ifit3*), transcription factors and signal transduction (e.g., *Pou2f2*, *Il10rb*, *Il15*), cell cycle, apoptosis, and DNA repair (e.g., *Eif2ak2*, *Pml*), and metabolism and miscellaneous functions (e.g., *Lacc1*, *Apoe*). These downregulated DEGs suggested weakened pathogen sensing and reduced IFN response or inflammatory signaling, indicating a potential shift to an anti-inflammatory phenotype in *Mtb*-infected BMMs of *Mafb*-cKO mice. KEGG pathway enrichment analysis revealed that oxidative phosphorylation and chemical carcinogenesis-ROS were enriched in the upregulated DEGs (**Figure 4A**), whereas ECM-receptor interaction, lysosome, and endocytosis were enriched in downregulated DEGs (**Figure 4B**). As depicted in the lysosome pathway diagram, the proton pump ATPeV, which plays a critical role in lysosomal acidification, is downregulated (**Figure 4C**) (33). We validated the expression of DEGs associated with selected GO terms in BMMS by qRT-PCR (**Figure 5**). As expected, *Cd74*, *H2-Ab1*, *Mmp12*, and *Nnt* were upregulated, whereas, *Ccl2*, *Gas6*, and *Ifit3* were downregulated in BMMs from *Mafb*-cKO mice relative to controls.

By GSEA using all expressed genes, a pathway of immune response to TB (https://www.wikipathways.org/instance/WP4197) exhibited impairment in *Mtb*-infected *Mafb*-cKO BMMs (**Supplementary Figure 2**). These enriched GOBPs and pathways are consistent with the previous results obtained from mRNA-seq of *Mtb*-infected *MAFB*-KD macrophages (10). IFN-gamma inducible chemokines (*Cxcl11*, *Ccl2*, *Ccl7*, *Cxcl9*, *Cxcl10*) were downregulated in *Mtb*-infected *MAFB*-KD macrophages, as well as in *Mtb*-infected BMMs from *Mafb*-cKO mice (**Supplementary Figure 3**). Thus, the regulation of gene expression by *Mafb* in mouse BMMs resembles that in PMA-stimulated human THP-1 macrophages during *Mtb* infection (**Supplementary Figure 4**).

### *Mafb* deficiency in macrophages increased mortality during *Mtb* infection in mice

**Figure 6:**
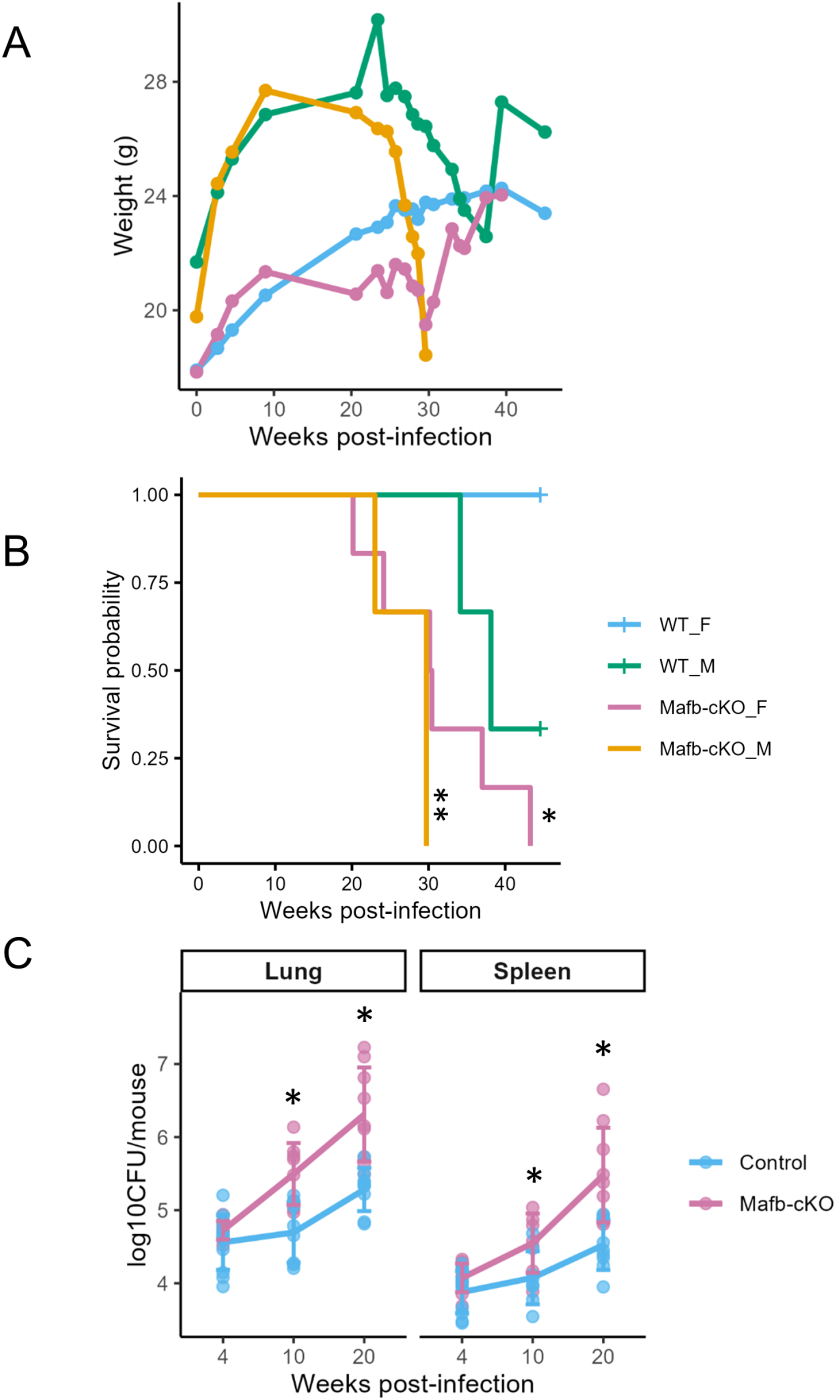
Effect of macrophage-specific *Mafb* deficiency on TB susceptibility in mice. **(A, B)** Body weight and survival of *Mafb*-cKO mice during *Mtb* infection. *Mafb*-cKO mice and wild-type (WT) mice (n = 9 per group) were aerosol- infected with *Mtb*, and their body weight **(A)** and survival **(B)** were monitored for 315 days. Survival probability between the two groups was analyzed by Kaplan–Meier analysis and the log-rank test. The median survival of female and male *Mafb*-cKO mice was 212 days (*P = 5 × 10^-4^, n = 6) and 208 days (**P = 0.03, n = 3), respectively, both significantly shorter than that of WT mice (267 days and 315 days, respectively). **(C)** Bacterial loads in the lungs of *Mtb*-infected *Mafb*-cKO and control mice were determined by CFU at 4 weeks, 10 weeks, and 20 weeks p.i (n = 8–11 per group at each time point). Data from individual mice is shown. *P < 0.01 using Tukey–Kramer test.

To examine whether *Mafb* deficiency in macrophages influences the outcome of *Mtb* infection in mice, we conducted aerosol infections in *Mafb*-cKO mice and WT mice and monitored them for 45 weeks (**Figure 1B**). *Mafb*-cKO mice began losing body weight and showed mortality starting at 20 weeks; by the end of the study, none remained alive (**Figures 6A, B**, **Supplementary Figure 5**). The survival probability was compared between groups of the same sex using Kaplan–Meier analysis and the log-rank test. The median survival of female (n = 6) and male (n = 3) *Mafb*-cKO mice was 212 and 208 days, respectively, which was significantly shorter than that of WT mice. Notably, male mice were more susceptible to *Mtb* infection than females, exhibiting greater body-weight loss and reduced survival. We next determined the bacterial burden in the murine organs after *Mtb* infection. At 10 and 20 weeks p.i., *Mafb*-cKO mice exhibited significantly higher burden in the lungs (**Figure 6C**). The spleens of *Mafb*-cKO mice also showed a higher burden at 10 and 20 weeks p.i., demonstrating the involvement of *Mafb* in the control of the bacterial burden in the lung and spleen. These results indicate that *Mafb*-cKO mice are more susceptible to *Mtb* infection than control mice, consistent with the phenotype observed in BMMs.

### Transcriptomics of *Mtb*-infected *Mafb*-cKO mouse whole lungs

**Figure 7:**
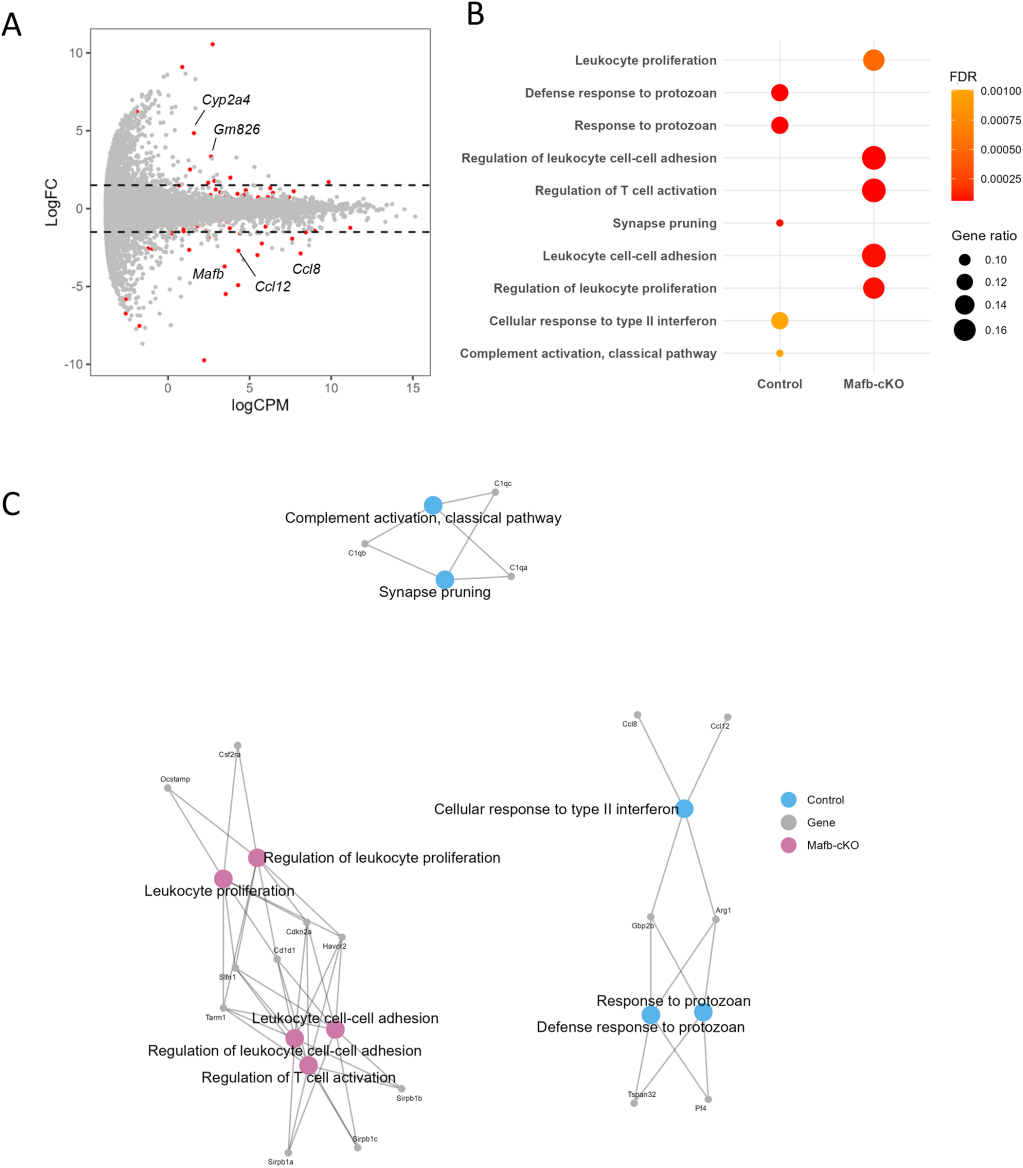
Transcriptomics of *Mtb*-infected *Mafb*-cKO mouse lung at 10 weeks p.i. *Mafb*-cKO mice and control mice were aerosol-infected with *Mtb* for 10 weeks (n = 6–10 per group). **(A)** MA plot showing 89 DEGs in *Mtb*-infected *Mafb*-cKO mouse lungs compared to those in *Mtb*-infected control mouse lungs, marked in red (FDR < 0.01). Each dot represents expressed genes in the sample. Log FC, log fold change. LogCPM, log count per million. **(B)** GO analysis for DEGs. Enriched GOBP categories in *Mtb*-infected *Mafb*-cKO lungs are shown. The color of each dot represents FDR, and the size represents gene ratio. **(C)** Gene concept network of the top 3 upregulated (Up) and downregulated (Down) GOBP categories in *Mtb*-infected *Mafb*-cKO mouse lungs. Upregulated GOBP categories include regulation of leukocyte cell–cell adhesion, regulation of T-cell activation, leukocyte cell–cell adhesion, and leukocyte proliferation, colored in salmon pink. Downregulated GOBP categories are defense response to protozoan, response to protozoan, cellular response to type II interferon, synapse pruning, and complement activation, and classical pathway, as shown in blue.

To investigate whether *Mafb* deficiency in macrophages alters BPs in the lungs during *Mtb* infection, we performed mRNA-seq on the whole lungs of *Mtb-*infected *Mafb*-cKO and control mice at 10 or 20 weeks p.i., respectively. At 10 weeks p.i., 89 genes were identified as DEGs in the lungs of *Mtb*-infected *Mafb*-cKO mice (**Figure 7A**, **Supplementary Table 2**). Among these 89 genes, 48 genes were upregulated and 41 genes were downregulated. GOBP of DEGs demonstrated that cell–cell adhesion, leukocyte proliferation, or the regulation of T-cell activation were activated, whereas complement activation, cellular response to type II IFN, synapse pruning, and response to protozoan were suppressed in the lungs of *Mafb*-cKO mice (**Figure 7B**, **Supplementary Table 4**). Concept gene network for GO categories visualized that *Cd1d1*, *Cdkn2a*, *Tarm1*, *Havcr2*, and *Slfn1* were the key genes for T-cell regulation (**Figure 7C**). KEGG pathway enrichment analysis demonstrated the enrichment of osteoclast differentiation in upregulated DEGs (**Supplementary Figure 6A**). Previous research demonstrated that MafB negatively regulates RANKL-mediated osteoclast differentiation (34). Consistent with this finding, downregulation of *Mafb* in our study led to upregulation of osteoclast differentiation–related genes (e.g., *Sirpb1c, Pira2, Sirpb1a*, and *Sirpb1b*). Complement components such as *C1qa*, *C1qb*, or *C1qc*, identified in suppressed GO categories, played a central role in complement activation. The involvement of *Mafb* in regulating complement components was consistent with the previous report (11). In addition to complement components, downregulated DEGs included cytokine ligands such as *Ccl8*, which is also known as *monocyte chemoattractant protein 2* (MCP2), *Ccl12*, known as *monocyte chemoattractant protein 5* (MCP5), or *Pf4*, known as *Cxcl4*. KEGG pathway enrichment analysis demonstrated that complement and coagulation cascade, and chemokine signaling pathway were enriched in downregulated DEGs (**Supplementary Figure 6B**).

**Figure 8:**
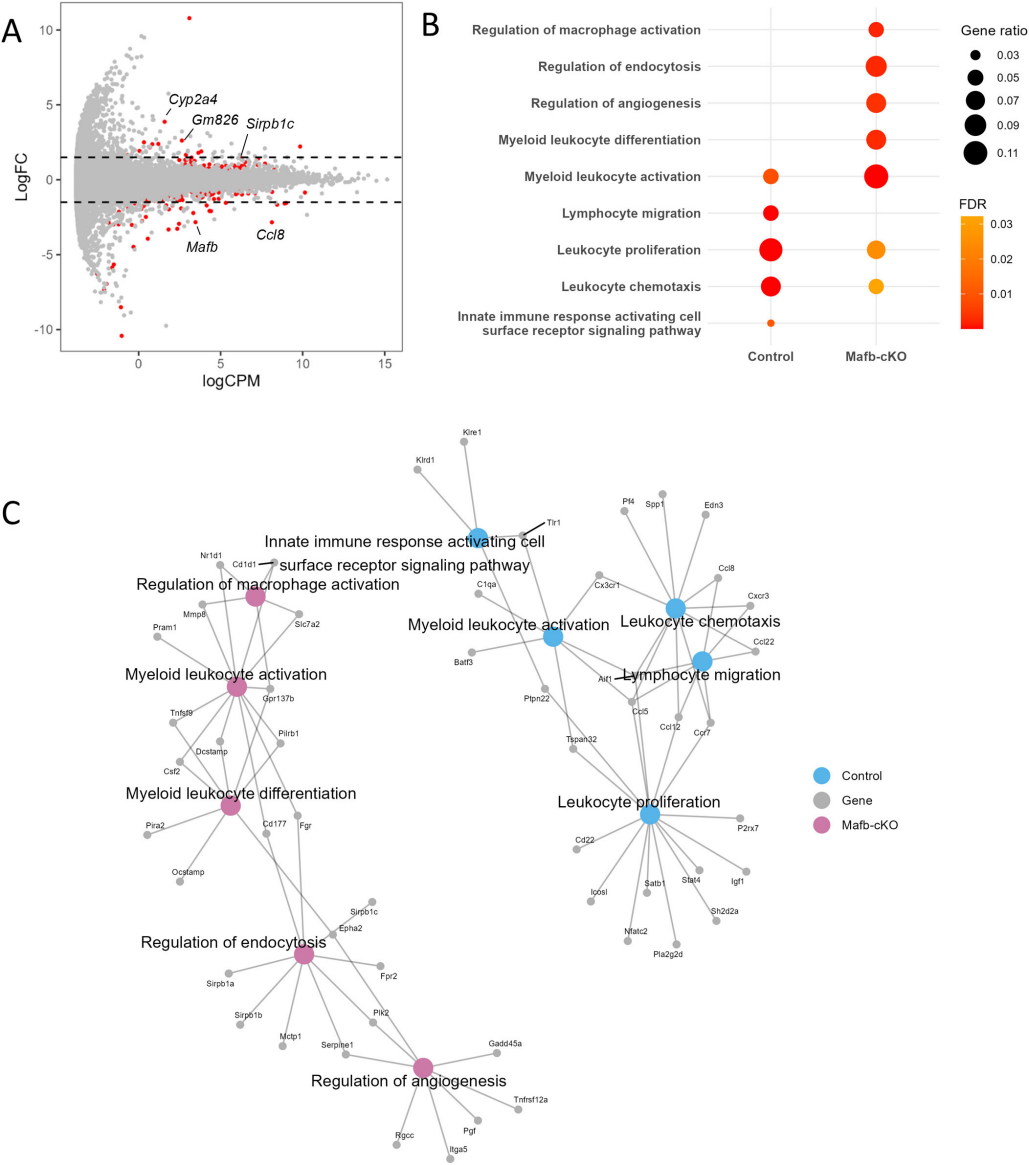
Transcriptomics of *Mtb*-infected *Mafb*-cKO mouse lung at 20 weeks p.i. *Mafb*-cKO mice and control mice were infected with an aerosol of *Mtb* for 20 weeks (n = 5–8 per group). **(A)** MA plot showing 267 DEGs in *Mtb*-infected *Mafb*-cKO mouse lungs in comparison with control mouse lungs marked in red (FDR < 0.01). Each dot represents expressed genes in the sample. Log FC, log fold change. LogCPM, log count per million. **(B)** GO analysis for DEGs. Enriched GOBP categories in *Mtb*-infected *Mafb*-cKO lungs were shown. The color of each dot represents FDR, and the size represents gene ratio. **(C)** Gene concept network of the top 3 upregulated (Up) and downregulated (Down) GOBP categories in *Mtb*-infected *Mafb*-cKO mouse lungs. The upregulated GOBP categories include myeloid leukocyte activation, regulation of endocytosis, myeloid leukocyte differentiation, regulation of angiogenesis, and regulation of macrophage activation colored in salmon pink. Downregulated GOBP categories are leukocyte proliferation, lymphocyte proliferation, immune response cell surface receptor signaling pathway, and leukocyte chemotaxis, shown in blue.

**Figure 9:**
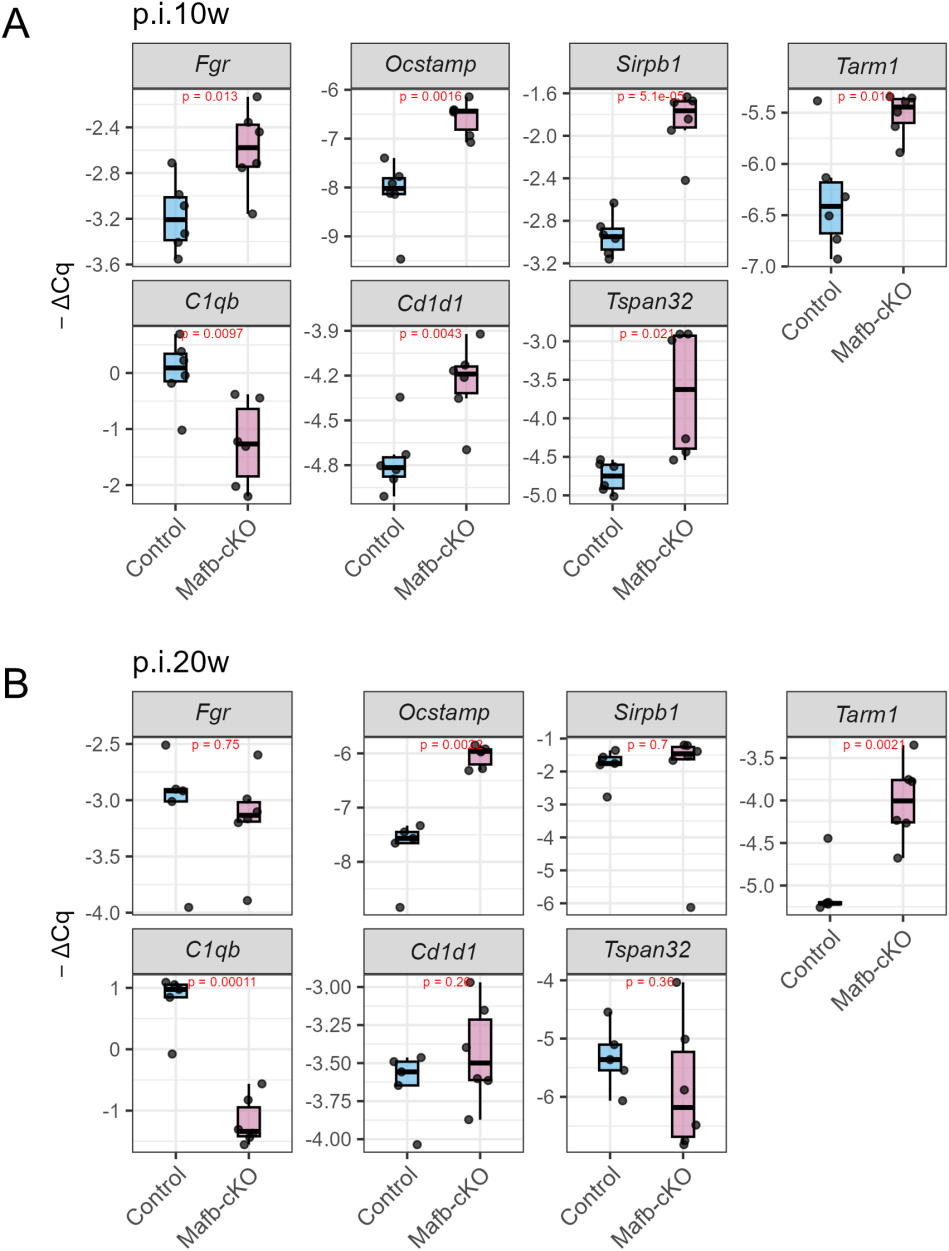
Validation of DEGs in Mtb-infected lungs at 10 p.i. **(A)** or 20 p.i. **(B)** by qRT-PCR. Seven representative DEGs were selected and validated by qRT-PCR (n = 5–6 per group at each time point). The upregulation (*Fgr, Ocstamp, Sirpb1, Tarm1*) or downregulation (*C1qb, Cd1d1, Tspan32*) of DEGs in Mafb-cKO lungs was confirmed by qRT-PCR.

As *Mafb*-cKO mice began to succumb around 20 weeks p.i., we also performed mRNA-seq to examine transcriptional changes in lungs between *Mafb*-cKO and control mouse. Differential expression analysis identified 267 DEGs (**Figure 8A**), of which DEGs found at 10 weeks p.i. were included. Among the 267 genes, 110 genes were upregulated and 157 genes were downregulated. GOBP showed that myeloid leukocyte activation, myeloid leukocyte differentiation, the regulation of macrophage activation, the regulation of endocytosis, and the regulation of angiogenesis were upregulated, whereas leukocyte migration, leukocyte chemotaxis, leukocyte proliferation, and immune response cell-surface receptor-signaling pathway were downregulated in the lungs of *Mtb*-infected *Mafb-*cKO mice (**Figure 8B**, **Supplementary Table 4**). Concept gene network revealed *Csfs* (GM-CSF), a key regulator for macrophage and dendritic cell function, *Mmp8*, *Cd177*, genes associated with neutrophil activation and migration, or *Sirpb1 family* for phagocytosis and immune modulation in upregulated DEGs, highlights strong differentiation and the activation of myeloid-derived immune cells (**Figure 8C**). Down regulated DEGs included *Ccl22*, *Ccl8*, *Ccl5*, *Cx3cr1*, *Pf4*, and *Ccr7*, which are involved in chemokine signaling or leukocyte migration; *P2rx7*, *Nfatc2*, and *Ptpn22*, regulatory genes in T-cell activation and immune tolerance, *Cd22* or *Icosl*, which are involved in B cell-mediated immune response, suggesting reduced adaptive immune activation and leukocyte or lymphocyte recruitment in the lungs of *Mtb*-infected *Mafb-*cKO mice compared to those of control mice (**Figure 8C**). We validated, by qRT-PCR, the expression of DEGs associated with selected GO terms in the lungs (**Figure 9**). While some genes (e.g. *Cd1d1* and *Tspan32*) showed results inconsistent with the RNA-seq data, others were consistent.

Since MafB is a transcription factor that binds Maf recognition elements (MAREs) in gene promoters (6), we evaluated whether DEGs in *Mtb*-infected BMMs and lungs from *Mafb*-cKO mice were subject to direct or indirect regulation by MafB. We compared our DEG sets with published MafB ChIP-seq data (24). Among the 1,223 DEGs in *Mtb*-infected *Mafb*-cKO BMMs, 413 (33.8%) overlapped with MafB-bound targets, including 175 upregulated and 238 downregulated genes (**Table 1**). In lungs from *Mtb*-infected *Mafb*-cKO mice, 28 DEGs (31.5%) at 10 weeks p.i. and 55 DEGs (20.6%) at 20 weeks p.i. overlapped with MafB-bound targets (**Table 1**). The proportion of DEGs directly bound by MafB was similar in *Mafb*-cKO BMMs and in lungs at 10 weeks p.i.; however, this proportion decreased at 20 weeks p.i., despite a greater number of DEGs overall. These findings suggest that secondary effects of *Mafb* deficiency contribute to the increased mortality observed in *Mafb*-cKO mice during the later stage of infection.

**Table 1:** Comparison of MafB ChIP-seq peaks and the DEGs.

Sample		Upregulated	Downregulated	Total
*Mafb*-cKO mouse BMMs	DEGs	614	609	1223
	MafB ChIP-seq peaks	175	238	413
		28.5%	39.1%	33.8%
*Mafb*-cKO mouse lungs, 10w	DEGs	48	41	89
	MafB ChIP-seq peak	14	14	28
		29.2%	34.1%	31.5%
*Mafb*-cKO mouse lungs, 20w	DEGs	110	157	267
	MafB ChIP-seq peak	24	31	55
		21.8%	19.7%	20.6%

MafB ChIP-seq peaks are compared with the DEGs identified in *Mtb*-infected *Mafb*-cKO mouse BMMs, *Mtb*-infected *Mafb*-cKO mouse lungs at 10 weeks p.i., or *Mtb*-infected *Mafb*-cKO mouse lungs at 20 weeks p.i. The ChIP-seq data (GEO GSM1964739/SRA SRX1465586) (24) was downloaded via ChIP-Atlas (accessed 27 June 2025) (25).

### Immune cell recruitment in the lungs of *Mtb*-infected *Mafb*-cKO mice

**Figure 10:**
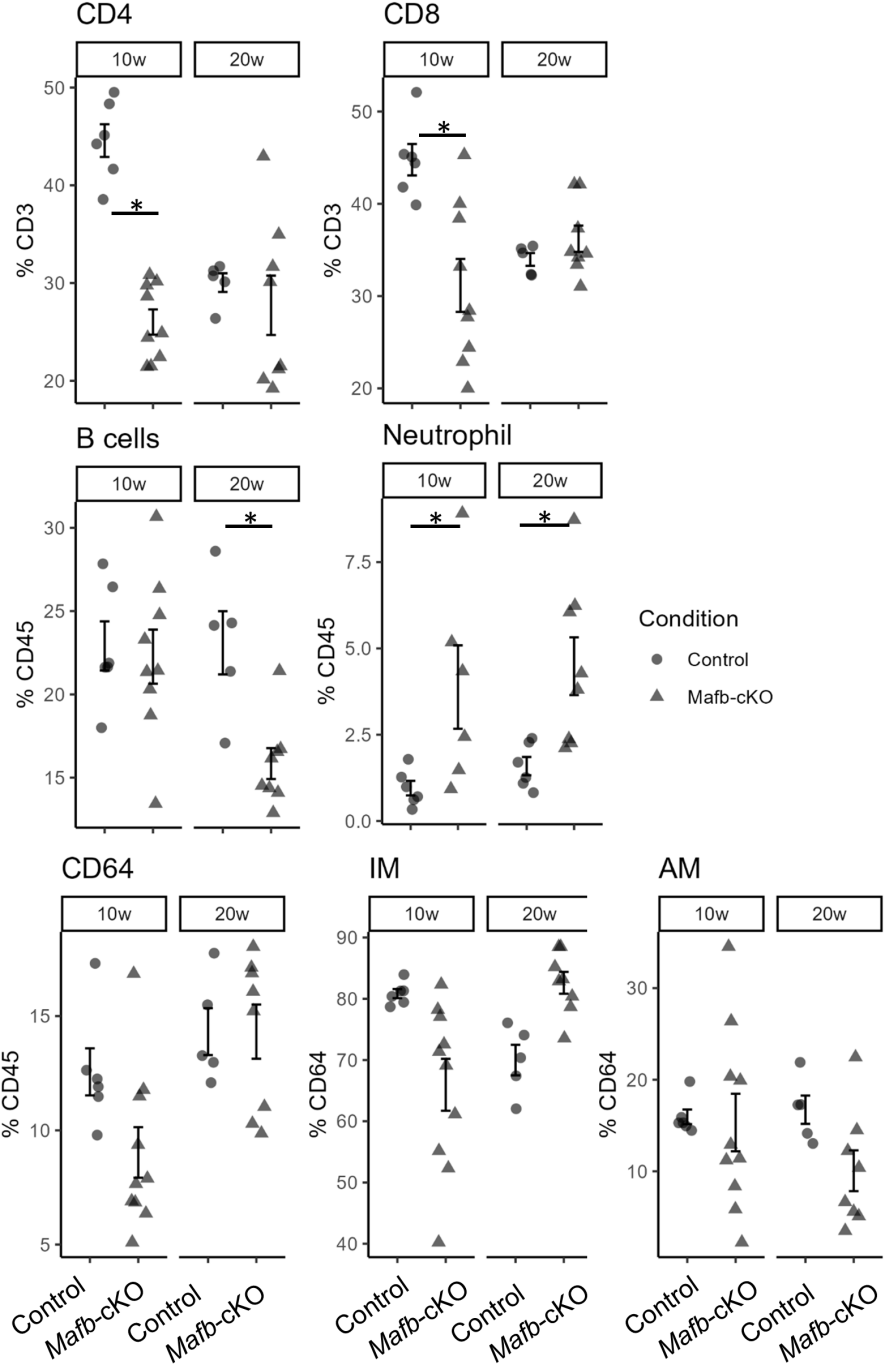
Population of immune cells in *Mtb*-infected *Mafb*-cKO mice. The proportion of the immune cell population in the lungs of *Mtb-*infected *Maf*b-cKO or control mice was determined by flow cytometry at 10 or 20 weeks p.i. (n = 5–10 per group at each time point of two experimental batches). The proportions of CD45^+^, B-cells, neutrophils, macrophages, CD4^+^ T-cells, CD8^+^ T-cells, and alveolar macrophages are shown. *P < 0.05 using the Wilcoxon test for each time point.

Transcriptomics of the lungs of *Mtb*-infected *Mafb*-cKO mice suggested altered recruitment of immune cells during *Mtb* infection (**Figures 7**, **8**). Therefore, we investigated the proportion of immune cells in the lungs of *Mafb*-cKO mice during *Mtb* infection by flow cytometry (**Figure 10**). The frequencies of both CD4^+^ and CD8^+^ T-cells were high at 10 weeks p.i., and then decreased at 20 weeks p.i. in control mice, whereas they were at the same levels in *Mafb*-cKO mice during infection, suggesting blocked early recruitment of CD4^+^ and CD8^+^ T-cells in the infected lungs of *Mafb*-cKO mice (**Figure 7**). The frequency of B-cells remained the same from 10 weeks to 20 weeks p.i. in the control mice; however, it decreased in *Mafb*-cKO mice at 20 weeks p.i., which supports the transcriptomics data. Despite the impaired chemokine signaling, the frequency of neutrophils was significantly higher in *Mafb*-cKO at 10 or 20 weeks p.i. compared to that in control mice. Although the frequency of interstitial macrophages was slightly lower in *Mafb*-cKO mice, the difference was not statistically significant, likely due to high inter-sample variability, which may explain the heterogeneity in disease development. Nonetheless, these results indicate that *Mafb* deficiency in macrophages affects the recruitment of various immune cells to the lungs of *Mtb*-infected mice.

We assessed the neutrophil recruitment by histpathological analysis (**Supplementary Figure 7**). On H&E staining, the whole lung architecture of control and *Mafb*-cKO mice at 10 weeks p.i. appeared similar. By immunohistochemistry for S100a9, a neutrophil marker, *Mafb*-cKO mice showed stronger S100a9 signals with more neutrophil infiltration into lymphocyte-rich granulomas than the control mice, consistent with the flow cytometric analysis (**Figure 10**).

## Discussion

To understand *MAFB*’s role in *Mtb* infection, we investigated its function using a murine model of myeloid-specific *Mafb* conditional-knockout (*Mafb*-cKO) mice. Transcriptomics of BMMs from *Mafb*-cKO mice revealed that a ROS metabolic process and oxidative phosphorylation were activated, whereas the IFN responses were suppressed during *Mtb* infection (**Figure 2**), which is consistent with our previous results (10). Therefore, we assume that *MAFB* in human and murine macrophages acts similarly in response to *Mtb* infection (**Supplementary Figure 4**).

We found that BMMs from *Mafb*-cKO mice failed to control intracellular *Mtb* proliferation (**Figure 2**). In fact, *Mtb* infection typically induces the production of ROS in infected macrophages to reduce the intracellular bacterial load (35). However, an imbalance between ROS and antioxidants leads to oxidative stress, which contributes to the onset and progression of TB (36, 37). In BMMs from *Mafb*-cKO mice, the ROS metabolic process was activated, and its related genes were identified. CYBB, CAMK2B, and ITPR1 are involved in ROS production, and SOD2, GPX3, and CAT are involved in ROS clearance (38). CYBB is the major catalytic subunit of nicotinamide adenine dinucleotide phosphate (NADPH) oxidase, encoding NOX2 that possesses antimicrobial activity against *Mtb* (39). The superoxide dismutase expressed by *Sod2* detoxifies the major ROS to protect host cells from the damage caused by excessive ROS. Paradoxically, the overexpression of *Sod2* promotes the intracellular survival of *Mtb* (40). During *Mtb* infection, genes related to both ROS production and clearance were upregulated in BMMs derived from *Mafb*-cKO mice, implying that BMMs from *Mafb*-cKO mice generate ROS to clear pathogen while maintaining redox balance to prevent self-damage during infection, thereby actively attempting to eliminate the excessive ROS.

*Mtb* infection also activates antiviral responses, including the induction of type I IFNs, in infected macrophages (41, 42). Type I IFNs can exacerbate disease, as shown by IFN-I–driven susceptibility in *Sst1*-sensitive mice (43). During infection, mycobacterial DNA is initially released from the phagosomes into the cytosols, where it is recognized by cyclic GMP-AMP synthase (cGAS), initiating type I IFN production. This recognition triggers the activation of the cGAS-STING-TBK1 cascade and transcription factors IRF3 and IRF7, followed by the production of type I IFNs and other cytokines (44). Activated IRF3 translocates into the nucleus and binds to IFN-stimulated response element (ISRE) in the promoters of type I IFNs and proinflammatory genes for further transcriptional induction (45). It has been shown that IRF3 is essential for downstream genes, such as *Cxcl10* and *Ifit1*, which are induced by IFN-β and IFN-γ (46). In *Mtb*-infected BMMs from *Mafb*-cKO mice, *Tbk1*, *Irf3*, *Irf7*, *Stat1*, *Stat2*, and other genes with ISRE were significantly downregulated (**Figure 3**), suggesting that *Mafb* regulates cGAS-STING-TBK1 cascade.

*Mtb*-infected BMMs from *Mafb*-cKO mice showed higher intracellular *Mtb* burden despite downregulation of type I IFN signaling. Pathway analysis suggested impaired lysosome biogenesis (**Figures 4**) and downregulation of TB pathway genes (**Supplementary Figure 2**), together with dysregulated ROS metabolism. These macrophage-intrinsic defects may contribute to the increased bacterial burden *in vitro*, independent of type I IFN signaling. Although type I IFN–related genes were reduced in *Mafb*-cKO BMMs upon *Mt*b infection, this change alone is unlikely to account for the increased intracellular bacterial proliferation observed in these macrophages. Excessive type I IFN signaling has generally been associated with detrimental outcomes during TB (43), and therefore reduced type I IFN activity would not be expected to promote bacterial proliferation. Rather, the impairment of lysosomal maturation and ROS homeostasis appears to override any potential effects of altered IFN signaling and likely represents the primary mechanism underlying the increased bacterial burden in *Mafb*-cKO BMMs. Additionally, genes related to the type II IFN pathway were also downregulated in *Mafb*-cKO BMMs during *Mtb* infection (**Supplementary Figure 3**), which may reflect secondary transcriptional changes associated with increased intracellular bacterial load. Thus, decreased IFN pathway activation in *Mafb*-deficient macrophages should be interpreted as a secondary consequence, rather than a major determinant of bacterial growth. *In vivo*, type II IFN-related transcripts were downregulated in the lungs of *Mtb*-infected *Mafb*-cKO mice at 10 weeks p.i. (**Figure 7**). These findings suggest that *Mafb* deficiency primarily compromises macrophage antibacterial functions, such as lysosome biogenesis and ROS homeostasis. Secondarily, uncontrolled bacterial proliferation in the organs may further contribute to disease progression at later stages.

Transcriptomics of the lungs of *Mtb*-infected *Mafb*-cKO mice displayed activated myeloid-derived immune cells and differentiation (**Figure 4**). This finding is consistent with the reports that low MafB levels activate self-renewal in resident macrophages (24, 47). Vanneste et al. exhibited that myeloid-specific *Mafb* deletion increased both the proliferative ability and cell death in macrophages, decreasing number of macrophages in the mouse lungs (48). We also demonstrated a slightly decreased population of CD64^+^ macrophages among immune cells at 10 weeks p.i. but not at 20 weeks p.i. (**Figure 10**). We displayed a significantly higher frequency of neutrophils in *Mafb*-cKO mouse lungs (**Figure 10**). The result is consistent with the necrosis of macrophages and bacillary replication induce neutrophil recruitment (49). In fact, neutrophil accumulation correlates with increased disease severity, suggesting that excessive neutrophils may exacerbate TB pathology (50). *Mtb*-infected *Mafb*-cKO mice displayed a higher bacterial burden at 10 weeks p.i. in the lungs. Neutrophil recruitment is further enhanced by their release of mediators in response to *Mtb* (51). These findings suggest that excessive neutrophil recruitment perpetuates inflammation and worsens TB pathology in *Mafb*-cKO mice. Kanai et al. also demonstrated an increased myeloid-cell infiltration, including neutrophils, in an ischemic acute kidney injury (AKI) model in *Mafb*-cKO mice, suggesting that *Mafb* is involved in myeloid-cell migration both in the site of infection and injury (52). Considering that *Mafb* regulates thermogenesis in brown adipose tissue in *Mafb*-cKO mice under cold conditions (53), it is suggested that *Mafb* controls various homeostatic functions in macrophages under infections, injuries, or cold conditions.

In *Mtb*-infected *Mafb*-cKO mice, the infiltration of mononuclear cells into the lungs showed substantial between-sample variability, whereas neutrophil infiltration in control mice exhibited relatively low (**Figure 10**). We interpret the increased variance in *Mafb*-cKO mice as a secondary effect: the knockout alters the tissue cytokine milieu (e.g., IFNs and chemokine gradients), leading to heterogeneous priming and state distributions of monocytes/macrophages across individuals. After controlling for technical covariates, the elevated variance persists, indicating that *Mafb*-cKO expands phenotypic heterogeneity in mononuclear phagocytes instead of enforcing a uniform transcriptional shift. To address whether *Mafb*-dependent transcriptional changes are direct or indirect (secondary), we overlaid out DEGs in *Mtb*-infected BMMs and lungs with published MafB ChIP-seq peaks (**Table 1**). As expected, DEGs in *Mtb*-infected BMMs and lungs at 10 weeks p.i. a substantial subset of transcriptional changes is consistent with direct MafB regulation. By contrast, in lungs at later stages, the transcriptome becomes increasingly dominated by indirect (secondary) networks. This framework also included IFNs-related genes (e.g., *Ifi202b, Gbp2b, Ccl5, Aqp4*).

We demonstrated that *Mafb* deficiency in macrophages impaired the cell signaling for leukocyte migration and the recruitment of CD4^+^ and CD8^+^ T-cells in the lungs of *Mtb*-infected mice, suggesting weakened adaptive immunity at an early stage of infection. Several studies have shown the importance of macrophage activation by IFN-γ produced from CD4^+^ T-cells for protective immunity against *Mtb* in mice (54–56). The depletion of CD4^+^ T-cells leads to increased bacterial loads and increased severity of the infection in *Mtb*-infected C57BL/6 mice (57). In a macaque model, CD4^+^ T-cells display an “innate-like” defense system and serve as master helper cells to recruit other Th-like effector cells, thereby successfully preventing early extrapulmonary *Mtb* dissemination (58).

In summary, the present study provides evidence that *Mafb* depletion in myeloid cells not only impairs macrophage bactericidal activity but also disrupts immune cell recruitment, leading to failed bacterial control and higher mortality in *Mtb*-infected mice.

### Limitations

Several limitations should be considered when interpreting our findings. Although *Mafb*-cKO BMMs exhibited significantly higher CFU at 7 days p.i., we did not observe a corresponding increase in cytotoxicity in the assay. This discrepancy may reflect differences in assay sensitivity, as the cytotoxicity assay predominantly measures cellular metabolic activity rather than direct cell death. Additionally, the medium change performed on day 3, required to maintain long-term cultures, may have influenced metabolic readouts and reduced the ability to detect subtle differences in viability at later time points. Using bacteria expressing a fluorescent protein, intracellular fluorescence signals did not reveal a clear difference between control and *Mafb*-cKO BMMs (**Supplementary Figure 8**), likely due to methodological limitations such as limited dynamic range, signal saturation, and inability to distinguish viable from nonviable bacteria. In contrast, CFU enumeration selectively quantifies viable replicating bacteria and is therefore more sensitive for detecting early differences in bacterial proliferation within macrophages. Although transcriptomic data indicated reduced expression of *Ccl2* and *Cxcl10* in *Mafb*-cKO BMMs during *Mtb* infection, the corresponding protein level measured by ELISA was not significantly different between groups (**Supplementary Figure 9**). Finally, we did not evaluate T cell activation in *Mafb*-cKO mouse lungs, and therefore our study cannot fully determine how *Mafb* deficiency influences the relationship between intracellular bacterial replication, macrophage death modalities, and downstream immune responses during *Mtb* infection.

## Data Availability

The datasets presented in this study can be found in online repositories. The names of the repository/repositories and accession number(s) can be found below: https://ddbj.nig.ac.jp/search/entry/bioproject/PRJDB20606.

## Supplementary material

Supplementary Figure 1 Integrated Genomics Viewer images show RNA-seq reads alignment at the *Mafb* and *Rplp1* genes in BMMs from control and *Mafb*-cKO mice. *Mafb*-cKO BMMs exhibited the loss of reads coverage on the *Mafb* gene, whereas control BMMs displayed the robust reads. Read coverage of *Rplp1*, a house keeping gene was comparable between control and *Mafb*-cKO BMMs.

Supplementary Figure 2 Gene set enrichment analysis was performed on all the genes expressed in *Mtb*-infected *Mafb*-cKO BMMs. Using the Wiki pathway, the immune response to tuberculosis (WP4197) was enriched. The pathway diagram and enrichment plot of the immune response to tuberculosis can be seen. Genes in the pathway are color-coded based on logFC in *Mtb*-infected *Mafb*-cKO BMMs compared to those in *Mtb*-infected control BMMs. Circles on the gene name represent elevated catalytic reaction. NES, normalized enrichment score.

Supplementary Figure 3 Heatmap of the z-score-ranked mRNA expression of five interferon-gamma inducible inflammatory chemokines (n = 4 per group). The gene expression of *Cxcl11*, *Ccl2*, *Ccl7*, *Cxcl9*, and *Cxcl10* was decreased in *Mtb*-infected *Mafb*-cKO BMMs.

Supplementary Figure 4 Summaries of transcriptomic and phenotypic responses to Mtb infection in PMA-stimulated *MAFB*-knockdown THP-1 cells (MAFB-KD macrophages), based on our previous study (10) **(A)**, and in BMMs from *Mafb*-cKO mice in the present study **(B)**.

Supplementary Figure 5 The body weight of each mouse was monitored during the survival challenge (male: n = 3 per group, female n = 6 per group). WT_F, female WT mice, WT_M, male WT mice, Mafb-cKO_F, female *Mafb*-cKO mice, Mafb-cKO_M, male *Mafb*-cKO mice. ^†^ indicates the time of death.

Supplementary Figure 6 KEGG pathway enrichment analysis was performed on the upregulated **(A)** and downregulated **(B)** DEGs of *Mtb*-infected *Mafb*-cKO mouse lungs at 10 weeks p.i. The color of each dot represents FDR, and the size represents gene ratio.

Supplementary Figure 7 Histopathological analysis of *Mtb*-infected lungs in *Mafb*-cKO mice. Representative H&E-stained whole-lung sections from *Mtb*-infected control **(A)** and *Mafb*-cKO mice **(B)**. Scale bar, 5 mm. Representative granulomas immunostained for S100a9, a neutrophil marker, in *Mtb*-infected control **(C)** and *Mafb*-cKO mice **(D)**. Scale bar, 250 μm. **(E)** Granuloma regions were selected and S100a9^+^ cells were detected using QuPath (29). The violin plot shows the proportion of S100a9^+^ cells among total cells within granulomas in *Mtb*-infected lungs from two control (n = 30 and 48 granulomas) and two *Mafb*-cKO mice (n =35 and 55 granulomas). **P* < 0.01 using Welch’s *t*-test.

Supplementary Figure 8 Fluorescent *Mtb* in BMMs. **(A)** BMMs from *Mafb*-cKO mice was infected with DsRed-expressing *Mtb* for 1, 3, or 7 days. Scale bar, 50 μm. **(B)** Quantification of fluorescent bacteria in BMMs. The proportion of infected cells relative to the total number of cells were measured (n > 30 fields). n.s., not significant by Wilcoxon test at each time point.

Supplementary Figure 9 BMMs from control or *Mafb*-cKO mice were infected with *Mtb*. MCP-1 (Ccl2) and IP-10 (Cxcl10) concentrations in culture supernatants were measured by ELISA (n = 5). n.s., not significant by Weltch’s *t*-test at each time point.
