# Supplementary figures and images for: Mafb deficiency in myeloid cells increases susceptibility to Mycobacterium tuberculosis infection in mice
Source: Front Immunol. 2026 Jan 23;16:1660933. doi: 10.3389/fimmu.2025.1660933 (PMC12876153; doi:10.3389/fimmu.2025.1660933)

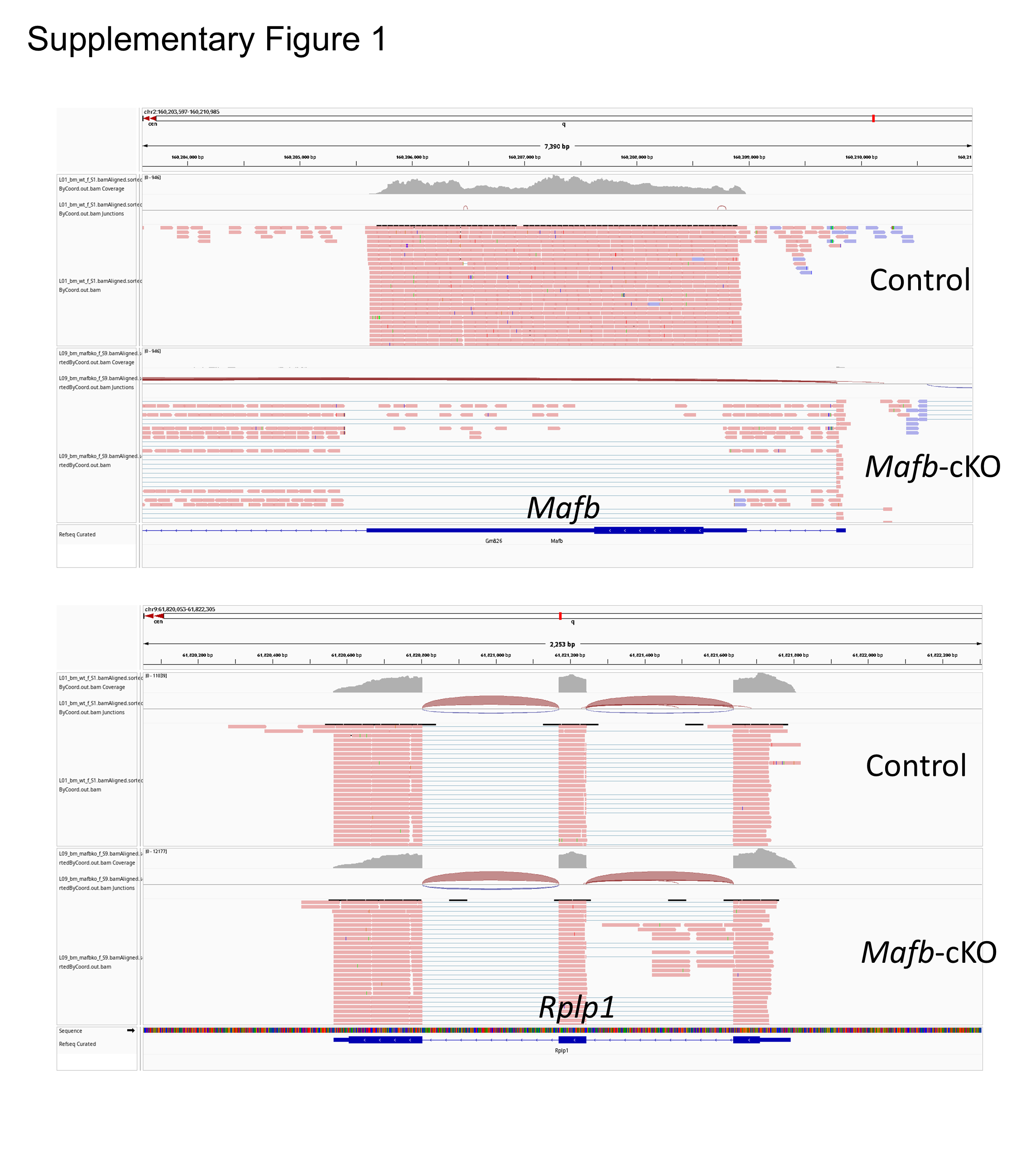

Supplement: Supplementary Figure 1 — Integrated Genomics Viewer images show RNA-seq reads alignment at the Mafb and Rplp1 genes in BMMs from control and Mafb-cKO mice. Mafb-cKO BMMs exhibited the loss of reads coverage on the Mafb gene, whereas control BMMs displayed the robust reads. Read coverage of Rplp1, a house keeping gene was comparable between control and Mafb-cKO BMMs. [file Image1.tif]

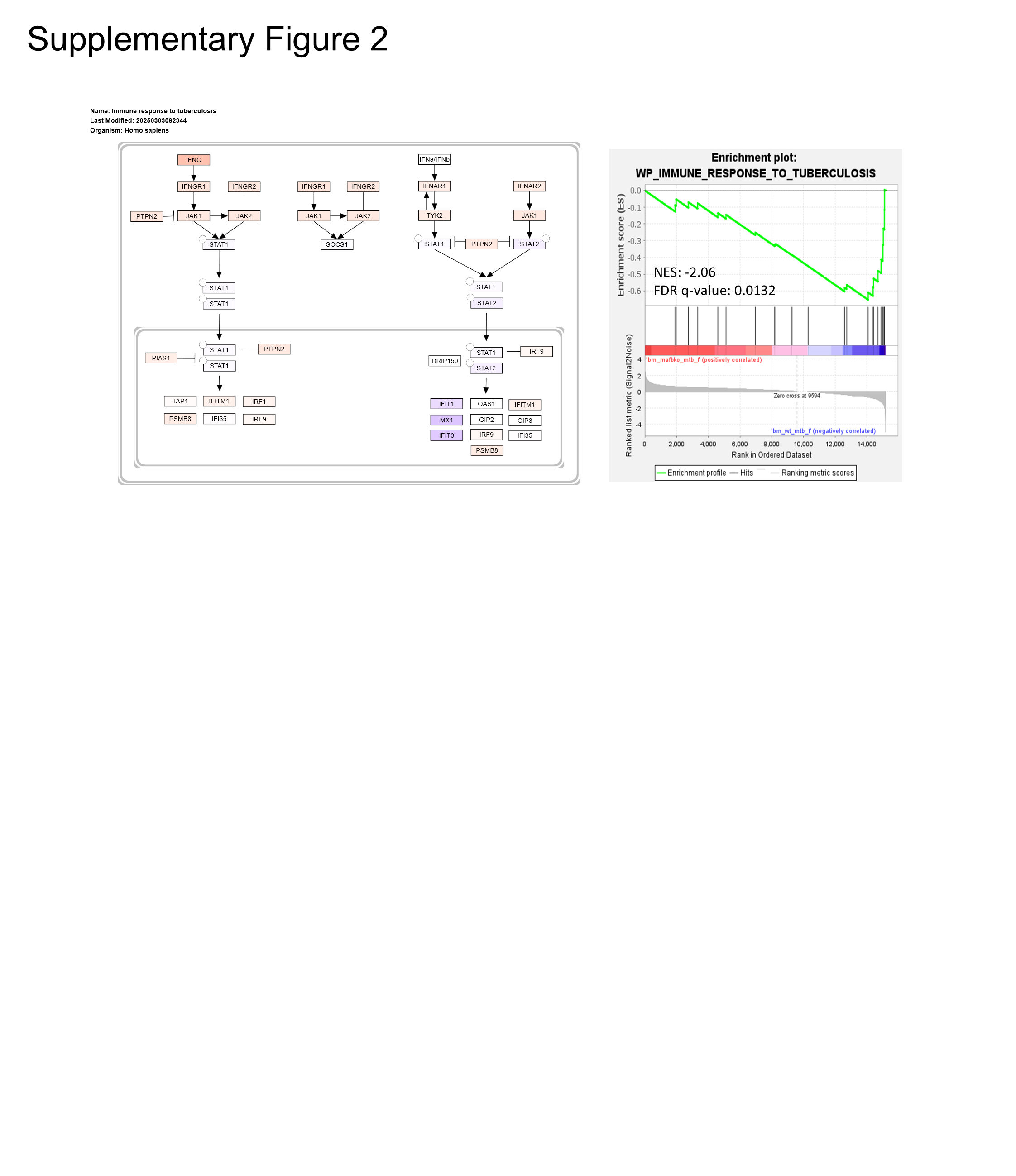

Supplement: Supplementary Figure 2 — Gene set enrichment analysis was performed on all the genes expressed in Mtb-infected Mafb-cKO BMMs. Using the Wiki pathway, the immune response to tuberculosis (WP4197) was enriched. The pathway diagram and enrichment plot of the immune response to tuberculosis can be seen. Genes in the pathway are color-coded based on logFC in Mtb-infected Mafb-cKO BMMs compared to those in Mtb-infected control BMMs. Circles on the gene name represent elevated catalytic reaction. NES, normalized enrichment score. [file Image2.tif]

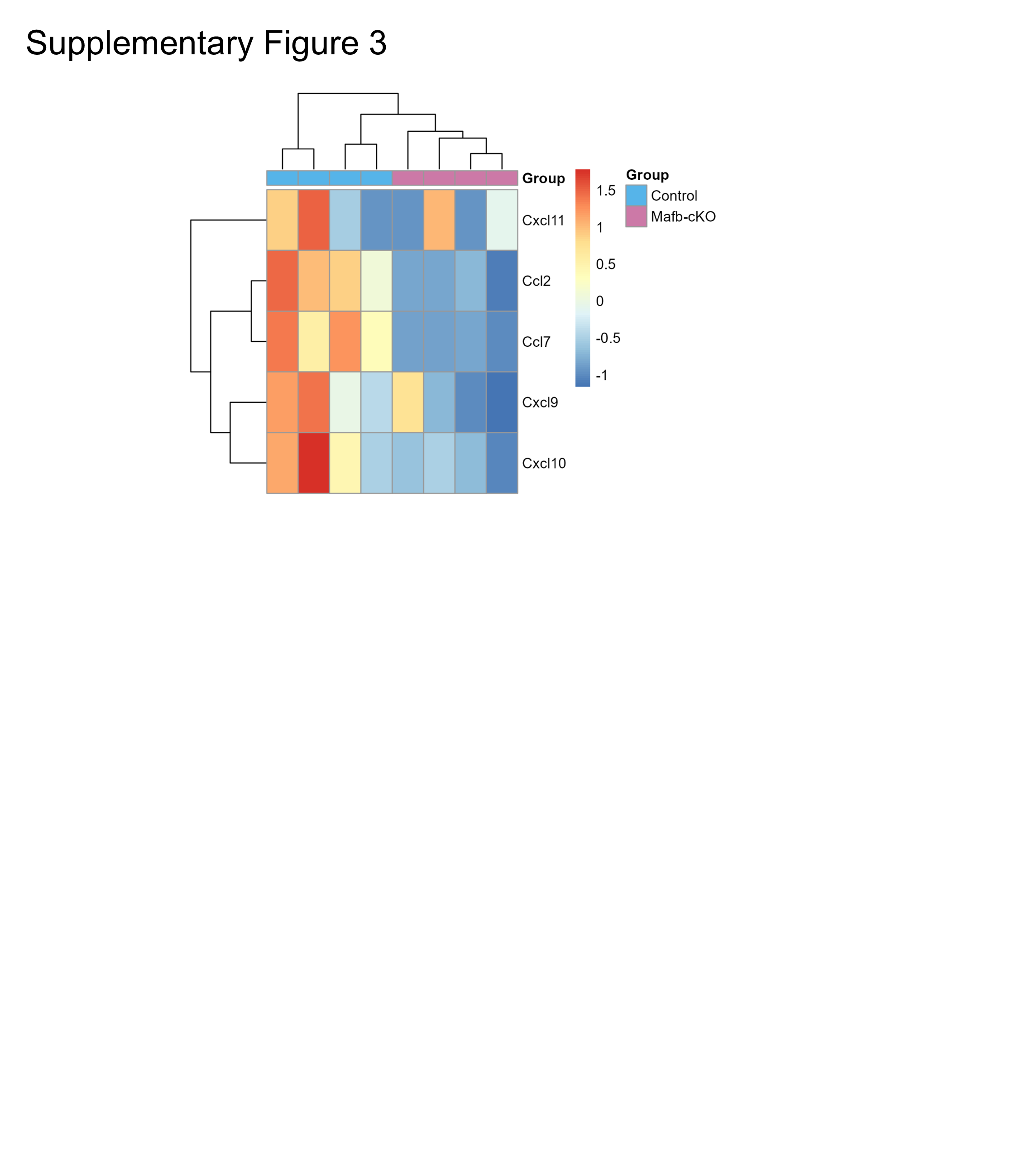

Supplement: Supplementary Figure 3 — Heatmap of the z-score-ranked mRNA expression of five interferon-gamma inducible inflammatory chemokines (n = 4 per group). The gene expression of Cxcl11, Ccl2, Ccl7, Cxcl9, and Cxcl10 was decreased in Mtb-infected Mafb-cKO BMMs. [file Image3.tif]

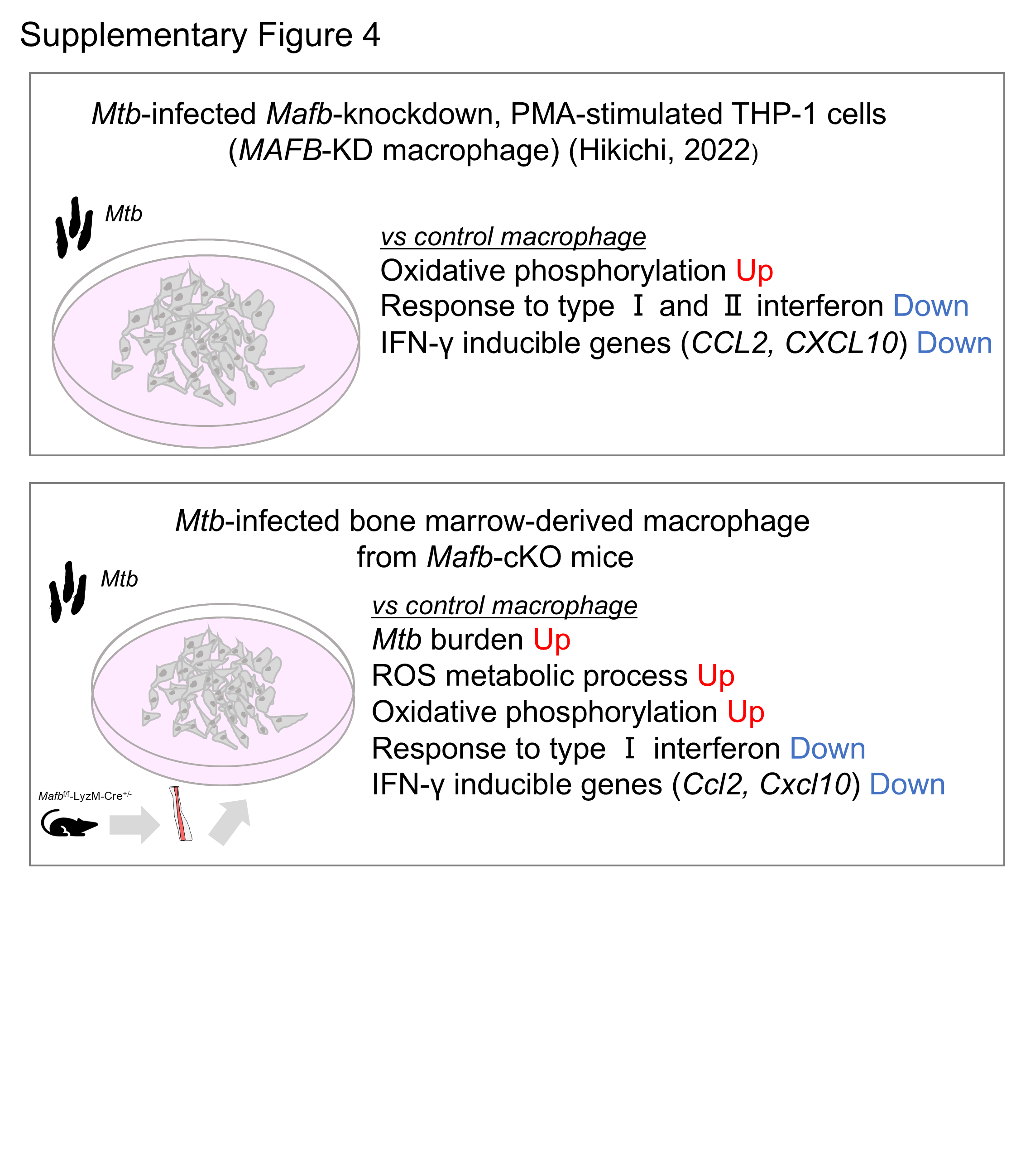

Supplement: Supplementary Figure 4 — Summaries of transcriptomic and phenotypic responses to Mtb infection in PMA-stimulated MAFB-knockdown THP-1 cells (MAFB-KD macrophages), based on our previous study (10) (A), and in BMMs from Mafb-cKO mice in the present study (B). [file Image4.tif]

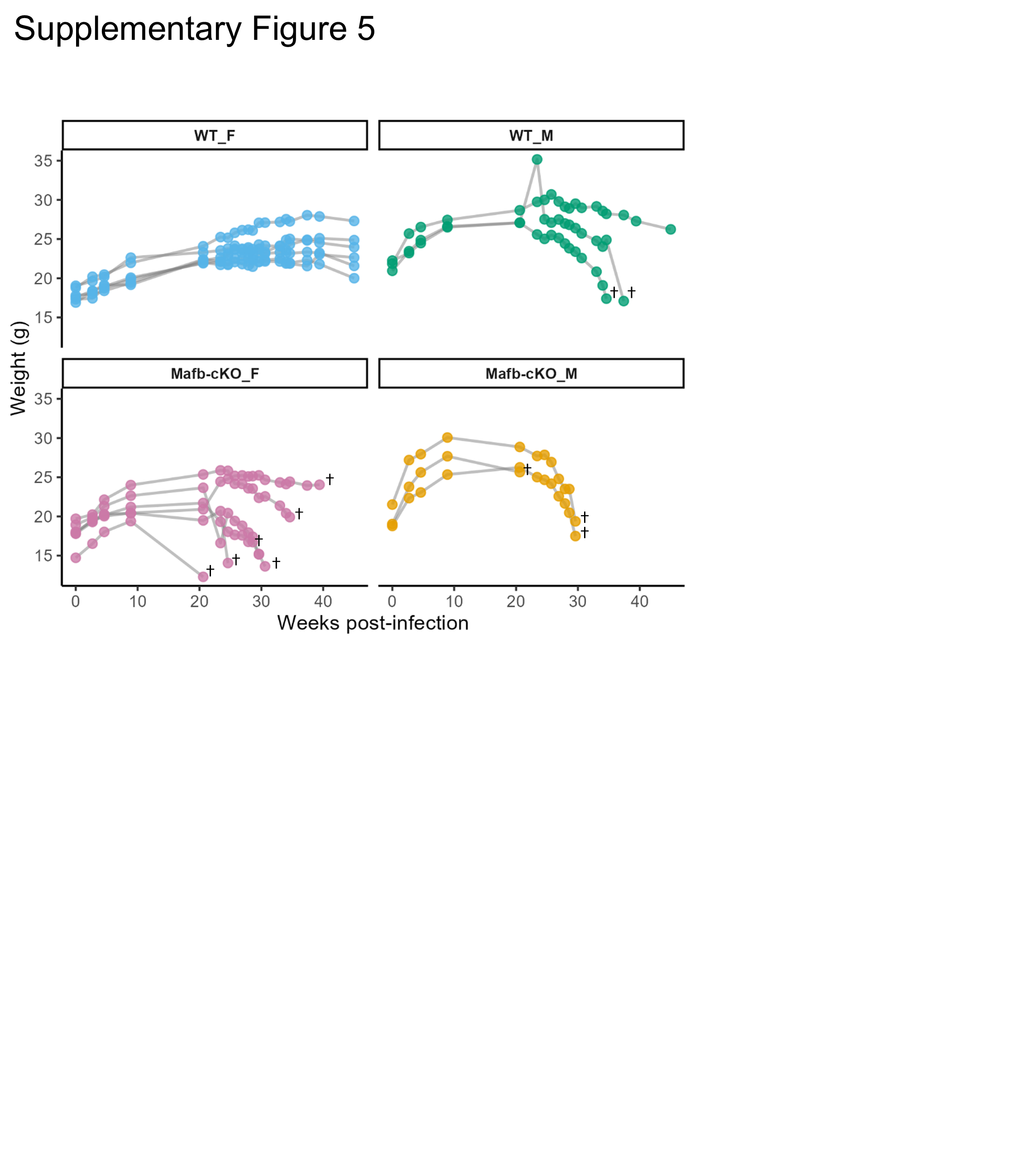

Supplement: Supplementary Figure 5 — The body weight of each mouse was monitored during the survival challenge (male: n = 3 per group, female n = 6 per group). WT_F, female WT mice, WT_M, male WT mice, Mafb-cKO_F, female Mafb-cKO mice, Mafb-cKO_M, male Mafb-cKO mice. † indicates the time of death. [file Image5.tif]

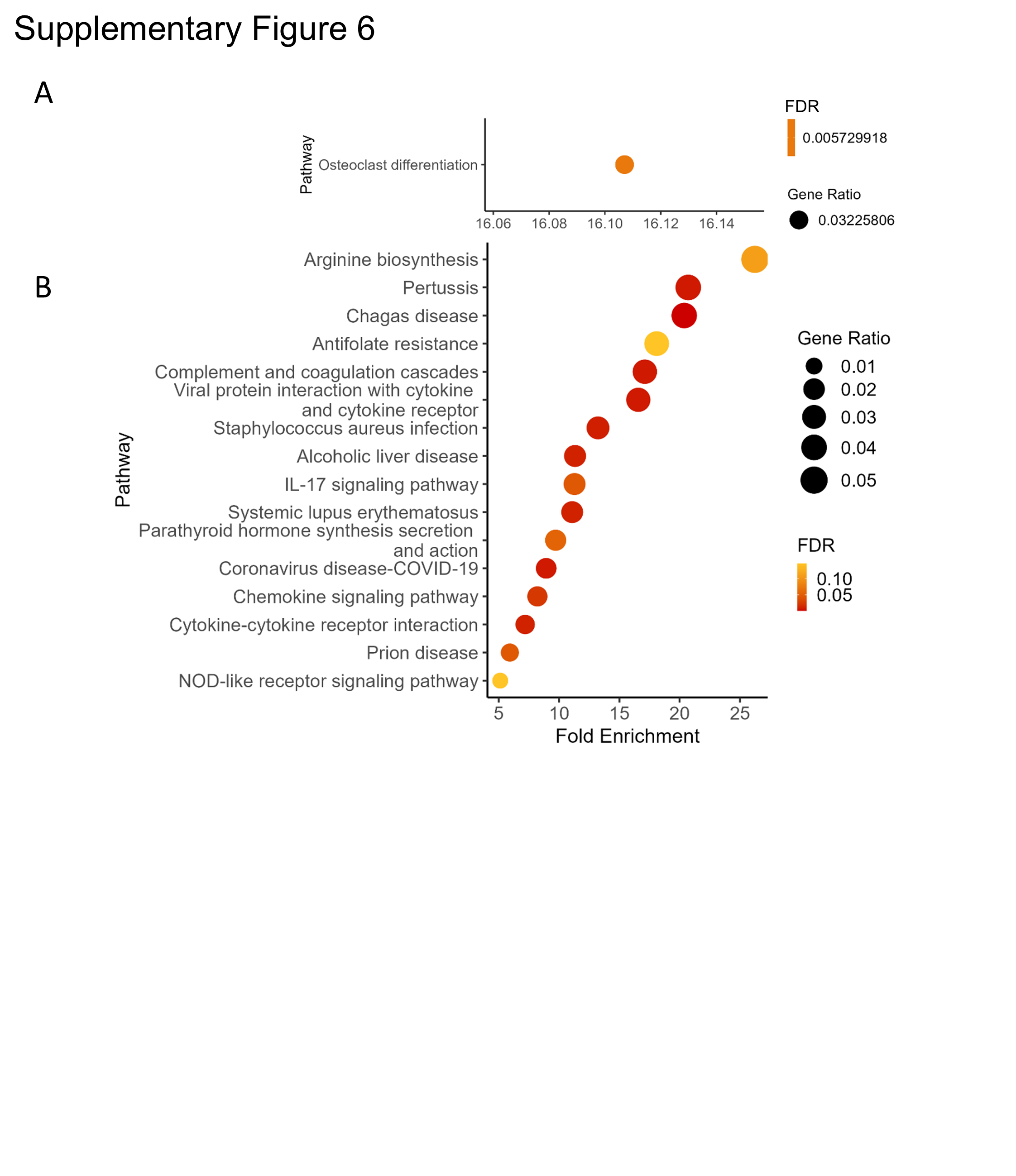

Supplement: Supplementary Figure 6 — KEGG pathway enrichment analysis was performed on the upregulated (A) and downregulated (B) DEGs of Mtb-infected Mafb-cKO mouse lungs at 10 weeks p.i. The color of each dot represents FDR, and the size represents gene ratio. [file Image6.tif]

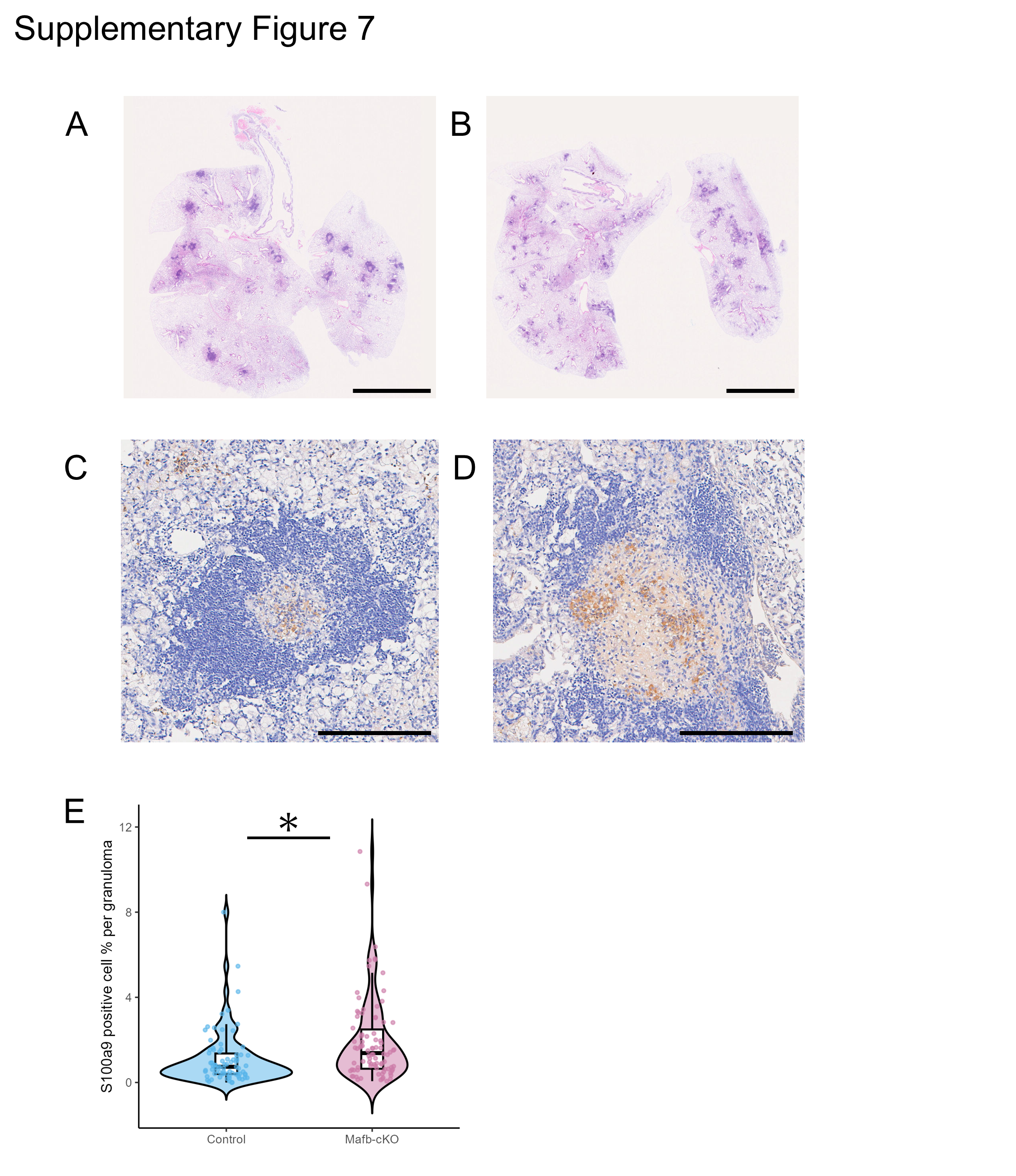

Supplement: Supplementary Figure 7 — Histopathological analysis of Mtb-infected lungs in Mafb-cKO mice. Representative H&E-stained whole-lung sections from Mtb-infected control (A) and Mafb-cKO mice (B). Scale bar, 5 mm. Representative granulomas immunostained for S100a9, a neutrophil marker, in Mtb-infected control (C) and Mafb-cKO mice (D). Scale bar, 250 μm. (E) Granuloma regions were selected and S100a9+ cells were detected using QuPath (29). The violin plot shows the proportion of S100a9+ cells among total cells within granulomas in Mtb-infected lungs from two control (n = 30 and 48 granulomas) and two Mafb-cKO mice (n =35 and 55 granulomas). *P < 0.01 using Welch’s t-test. [file Image7.tif]

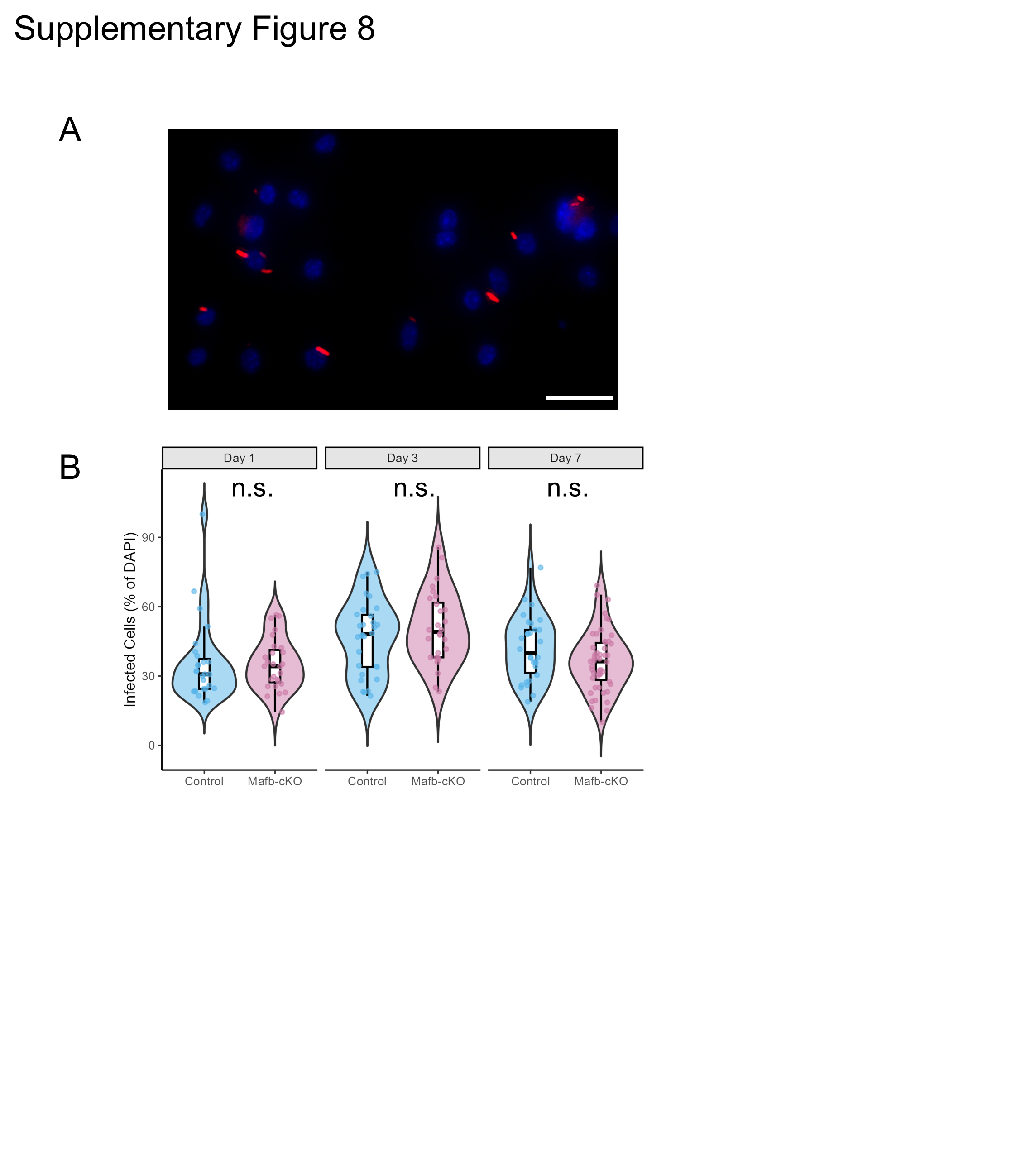

Supplement: Supplementary Figure 8 — Fluorescent Mtb in BMMs. (A) BMMs from Mafb-cKO mice was infected with DsRed-expressing Mtb for 1, 3, or 7 days. Scale bar, 50 μm. (B) Quantification of fluorescent bacteria in BMMs. The proportion of infected cells relative to the total number of cells were measured (n > 30 fields). n.s., not significant by Wilcoxon test at each time point. [file Image8.tif]

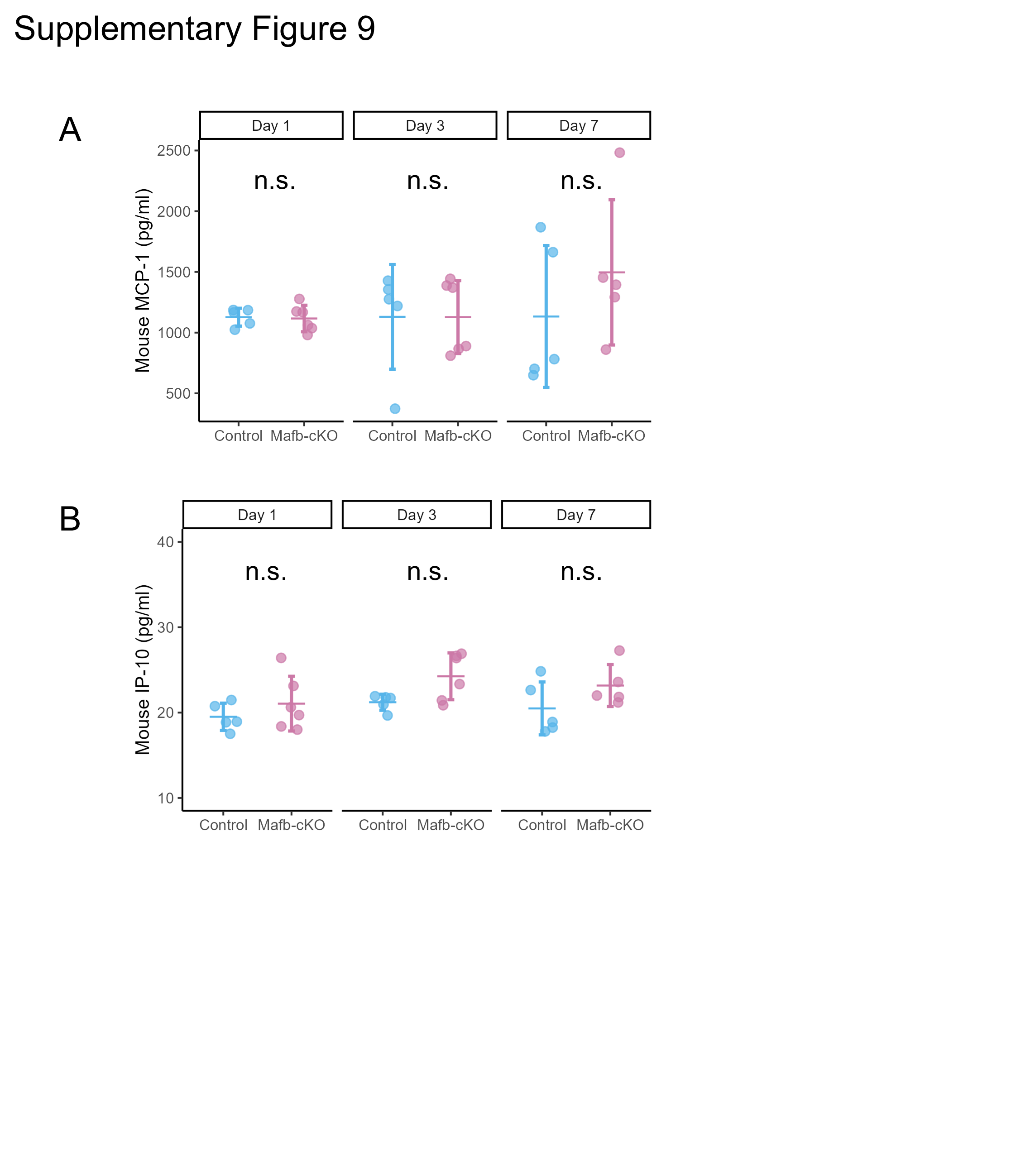

Supplement: Supplementary Figure 9 — BMMs from control or Mafb-cKO mice were infected with Mtb. MCP-1 (Ccl2) and IP-10 (Cxcl10) concentrations in culture supernatants were measured by ELISA (n = 5). n.s., not significant by Weltch’s t-test at each time point. [file Image9.tif]
